# Mindfulness-Based Cognitive Therapy in Clinical Practice: A Systematic Review of Neurocognitive Outcomes and Applications for Mental Health and Well-Being

**DOI:** 10.3390/jcm14051703

**Published:** 2025-03-03

**Authors:** Evgenia Gkintoni, Stephanos P. Vassilopoulos, Georgios Nikolaou

**Affiliations:** Department of Educational Sciences and Social Work, University of Patras, 26504 Patras, Greece; stephanosv@upatras.gr (S.P.V.); gnikolaou@upatras.gr (G.N.)

**Keywords:** mindfulness-based cognitive therapy (MBCT), mindfulness-based interventions (MBIs), subjective well-being, neuropsychological outcomes, emotional regulation, cognitive function, neuroplasticity, cognitive processes, neuroscientific models, psychological treatment

## Abstract

**Background/Objectives**: This systematic review outlines the neurocognitive outcomes and mechanisms of mindfulness-based cognitive therapy (MBCT) that influence subjective well-being. MBCT is a clinical intervention that integrates cognitive therapy with mindfulness practices to prevent depression relapses and improve mental health. **Methods**: The review focuses on the effects of MBCT on brain structure changes, cognitive processes, and emotional regulation, which are related to improvements in subjective well-being. A total of 87 studies were included in the review to assess the effectiveness of MBCT. **Results**: Evidence from the studies highlights the effectiveness of MBCT in reducing symptoms of depression, anxiety, and stress. MBCT was also shown to enhance cognitive functions and emotional regulation across diverse populations. These findings point to the potential for MBCT to induce neuroplastic changes in the brain and widen the applicability of the treatment for a variety of disorders, calling for further research into long-term benefits and underlying neurobiological mechanisms. **Conclusions**: The review emphasizes the potential of MBCT to bring about neuroplastic changes, calling for further research into its long-term benefits and the underlying neurobiological mechanisms. This study underlines the need to incorporate multidisciplinary measures by integrating psychology and neuroscience to comprehend comprehensively the effects of MBCT.

## 1. Introduction

Mindfulness-based cognitive therapy (MBCT) is a therapeutic approach that integrates elements of cognitive therapy with mindfulness practices. Initially developed by Zindel Segal, Mark Williams, and John Teasdale, MBCT was designed to prevent relapses in individuals suffering from recurrent depression by teaching them skills to recognize and counteract cognitive patterns that can lead to depressive episodes. Over the past two decades, MBCT has garnered significant attention for its effectiveness in not only preventing depression relapses, but also in improving a broad spectrum of mental health outcomes.

In the last two decades, its application has broadened, showing efficacy in attenuating symptoms of depression and anxiety, improving emotional regulation, and enhancing general psychological well-being. Depression and anxiety are clinically established mental disorders with clear diagnostic criteria and symptomatology. Anxiety disorders are defined by excessive worry, fear, and physiological hyperarousal, whereas depressive disorders are characterized by low mood, anhedonia, and cognitive impairments. MBCT is aimed at these disorders by educating subjects to detach from negative thought patterns, thus decreasing ruminative thinking and strengthening psychological resilience. Acute stress as a normal physiological reaction to adversity is different from chronic stress as a risk factor for depression and anxiety [1,2]. Chronic stress also changes neurobiological processes, such as dysregulation of the hypothalamic-pituitary-adrenal (HPA) axis and structural changes in the prefrontal cortex and amygdala, brain regions involved in emotional dysregulation, and cognitive impairment in mood disorders. How MBCT affects these processes can give us an understanding of how it works in enhancing mental health. The efficacy of MBCT goes beyond symptom change to more generalized neuropsychological effects. Neuroscience advances demonstrate that MBCT enhances neuroplasticity, recruiting brain regions subserving emotional processing, cognitive control, and self-awareness. For example, MBCT has been linked to increased prefrontal cortex activation, decreased amygdala reactivity, and enhanced connectivity in neural networks underlying cognitive and emotional regulation. These influences speak to the impact of MBCT on acquiring adaptive cognitive and emotional functioning and, ultimately, subjective well-being.

At its core, MBCT trains individuals to cultivate mindfulness, an active and open attention to the present moment. This mindfulness practice is characterized by a non-judgmental awareness of one’s thoughts, feelings, and bodily sensations. By fostering mindfulness, MBCT helps individuals develop a different relationship with their thoughts and emotions, reducing the tendency to ruminate and increasing emotional regulation. The mindfulness component of MBCT draws on techniques from mindfulness-based stress reduction (MBSR), another well-established mindfulness intervention developed by Jon Kabat-Zinn [3,4]. Mindfulness-oriented meditation has become increasingly popular in Western contexts as a form of mental training and well-being promotion, and mindfulness-based training programs have become widespread. Such programs are rooted in Buddhist traditions, but the teaching and the scientific conception of the mechanisms underlying their success have been transformed into a secular version. The notion of “mindfulness” has its roots in the Theravada tradition of Buddhism, where “sati”, “sampajanña”, and “upekkhā” for meditation refer to the four applications of mindfulness: awareness of the body, awareness of the felt states or sensations, mindfulness of mental factors or mental states, and mindfulness of mind-objects. Hospers, Janssen, and Chung have reported that, metanalytically, well-being is predicted most by universal measures of mental training outcomes such as meditation expertise, but least by contemporary meditation outcomes such as the witnessing level of ego development [5,6,7].

The origination of subjective well-being research and the subjective well-being of meditation experts is interesting. Several insights into this relationship are described, shedding light on the possible psychological mechanisms underlying this effect. MBCT is a treatment intervention that is successful in preventing recurrent depression and treating other recurrent depressive patients. The underlying cognitive processes of an increased risk of relapse for people with a history of three or more depressive episodes are suggested, such as a tendency to allocate attention to negative moods and units but less automatic vigilance of positive experiences [8,9,10,11]. Recent advances in neuroscience have enabled researchers to investigate the neuro-psychological effects of MBCT, thereby facilitating the necessary enlightenment on how this treatment makes a difference regarding brain structure and function. Neuropsychological outcomes are the changes in the brain that affect cognitive functions such as attention, memory, executive functions, and emotional regulations, according to [12,13]. This is rather important because of its provision for a biological basis to understand the effectiveness of MBCT. MBCT has been shown to bring about changes in the structure of brain regions, including the prefrontal cortex and amygdala, that are implicated in emotional regulation and functional changes in the support of cognitive processes by brain networks.

Other important indicators of mental health include objective well-being, which is a multivariate aspect that entails life satisfaction, the presence of positive emotions, and the absence of negative emotions. Enhancing subjective well-being is a central goal of many therapeutic interventions, including MBCT. Understanding how MBCT influences subjective well-being via neuropsychological changes provides deeper insights into its mechanisms of action. For example, improvements in emotional regulation and cognitive function resulting from MBCT will likely contribute to increased life satisfaction and reduced emotional distress. One principal purpose of the present study is to provide a detailed and comprehensive overview of the ideal selection of measures to include both psychology and neuroscience approaches in research. Within the MBCT programs, changes to sensory awareness are likely to have exclusive relevance regarding the degree to which abstract cognitive representations hijack sensory inflow that might originate automatic biological processes in the first place. Neural underpinnings of attribute transfer in emotional perception were possessed during the three successive brief periods after physiological arousal [14,15,16].

Despite the growing body of research on MBCT, there is a need for a comprehensive synthesis of findings related to its neuropsychological outcomes and their impact on subjective well-being. This systematic review aims to address this gap by examining the existing literature on the neuropsychological effects of MBCT and how these effects correlate with improvements in subjective well-being. By analyzing studies investigating changes in brain structure and function, cognitive processes, and emotional regulation, this review will provide a holistic understanding of the pathways to which MBCT enhances mental health.

## 2. Literature Review

Nowadays, mindfulness-based interventions’ claims of effectiveness are often related to inner states of well-being and emotion regulation. While most psychological states are hard to quantify, neuroscientific research has attempted to understand how they are being translated into neurobiological correlates. Neuroscientific methods such as heart rate variability (HRV), electroencephalography (EEG), and functional magnetic resonance imaging (fMRI) parameters should be combined with psychological testing in neuroscience research. The aim is to provide a detailed and comprehensive overview of the ideal selection of measures to include both psychological and neuroscience approaches in research. Within the MBCT programs, changes to sensory awareness are likely to have exclusive relevance regarding the degree to which abstract cognitive representations hijack sensory inflow that might originate automatic biological processes in the first place. Neural underpinnings of attribute transfer in emotional perception were possessed during the three successive brief periods after physiological arousal [14,15,16].

### 2.1. Definition of Mindfulness-Based Cognitive Therapy (MBCT)

MBCT is an 8-week, skills-based program that teaches the practice of mindfulness and a new way to relate to negative emotions. Derived from Jon Kabat-Zinn’s mindfulness-based stress reduction (MBSR), MBCT provides an approach that can help prevent people who have experienced major depression from falling back into depression symptoms. As a relapse prevention intervention, MBCT addresses negativistic processing styles and modular components of mindfulness that contribute to the initiation and perpetuity of depression rumination, mood-related avoidance, and suppression, as well as worry about future depressive episodes. In contrast to cognitive therapy for depression, which addresses themes and attitudes related to depression through talk therapy, MBCT formalizes skill-building, commonly applied cognitive therapy, with childlike curiosity and a presence of awareness to support progress in the cognitive restructuring of Bower’s negative cognitive triads. It also enhances positive, negative, and neutral affective bias abilities in semantic memory task performance, gap detection of emotional attention influence, and controls autonomic arousal [11,12,17,18].

### 2.2. Importance of Subjective Well-Being

Subjective well-being (SWB) has recently been an increasing focus of public and scientific attention because it is considered worldwide by researchers, politicians, and organizations aware of its importance. It is important to live a life marked by happiness, free of psychological disorders, and with hope for future projects. It is also essential to consider the negative consequences when SWB experiences a decrease. Depression is currently the leading cause of disability internationally, and its incidence rate is anticipated to increase [19,20,21,22,23].

Conversely, happiness, which implies a general sense of life enjoyment, optimism, and positive feelings, and life satisfaction, which means a global judgment of life quality considering the individual’s standards or values and current life circumstances, are frequently associated with SWB. They can buffer against demographic and economic factors, including education level, socioeconomic status, and employment, geographic location, among others, against the onset of many known chronic diseases: mortality, hypertension, obesity, diabetes, sleep disturbances, cancer, coronary artery disease, and several other physical conditions. They can also delay the pace of biological aging according to subjective age, the age individuals feel, not their chronological age [24,25].

### 2.3. Neuropsychological Foundations of Subjective Well-Being

The term SWB describes a person’s interpretation of their physical and mental state, affecting an individual’s evaluation of the quality of their life. It involves assessing affective components such as mood and emotions, cognitive components such as life satisfaction and domain-specific satisfaction with work, financial circumstances, and relationships, and a global assessment of these domains. Although the concept is essentially subjective in nature, the only reliable source of information on it is the self-assessment of the individual—SWB has several neurobiologically based and, thus, objectively measurable components. Consequently, a growing body of research in psychology and neural science has explored the neural systems underpinning SWB to enhance the clinical potential of methods that can ultimately improve SWB based on a neurobiological understanding of subjective experience. The neural systems underpinning SWB may help to detect possible relations between neuropsychological findings and clinical outcomes in mindfulness-based therapy (MBT) research [26,27].

Knowledge about these elements of SWB accounts for the escalation of training and exercises in psychological and cognitive therapies, which MBT is based on. The motivational and attentional aspects of SWB are considered, the main points of attaching SWB to these functions in the context of the models, and the stages in the MBT protocol and exercises are presented. These steps contain training and exercises focused on the micro and macro model processes of selective attention and regulation of emotions. This section does not discuss the activation of temporal and frontal areas and the implementation of reference-point-related decision-making. However, data on the neurobiological foundations of choice-happiness congruence and appreciation of these models will be generated in future MBT studies or in neurobiological studies in the psychophysiological range of microcomputers and by us and other research groups. Finally, data related to optimal neuropsychological models and an exploration algorithm for SWB in the MBT research context are presented, and aspects of its synthesis are proposed. It requires additional data on the neurobiological bases of choice-happiness congruence [28,29].

### 2.4. Neural Correlates of Well-Being

High levels of life satisfaction, positive affect, and experiences of fulfillment and meaning in life characterize subjective well-being. According to the hedonic perspective, subjective well-being reflects the balance between positive and negative emotional experiences. Building on the affect balance model, well-being has become a focus of positive psychology, which aims to broaden the focus from pathological conditions and suffering to factors that enhance emotional experiences and life satisfaction. One widely accepted model of well-being is the PERMA model, which defines well-being as the balance between positive emotional experiences, engagement in activities that absorb one’s attention and provide fulfillment, positive social relationships, a sense of meaning and purpose in life, and a sense of achievement. The PERMA model, developed by Martin Seligman, encompasses a broad framework through which well-being can be conceptualized. First is Positive Emotion, which refers to the state of happiness, joy, and optimism. Then, there is Engagement, or completely being absorbed in activities where one finds interest. The third would be Relationship or stressing the importance of social connectivity. Meaningfulness refers to feeling a sense of direction and being related to something bigger than oneself. Lastly, Accomplishment focuses on attaining personal objectives and feeling successful. All these components combined contribute to a well-rounded, full life. The cross-cultural validity and generalizability of the PERMA model are assessed in several countries throughout the world. A significant goal of well-being research is to develop interventions that help individuals to enhance their well-being and to cope with adversities in the long run. However, knowledge about the effects and benefits of these interventions is still limited [30,31,32,33].

Despite the increasing research in the emerging field of well-being, the underlying neurobiological mechanisms of well-being are still not sufficiently known. Another significant body of literature addresses the impact of meditation practices, such as mindfulness meditation, on well-being. Mindfulness meditation is rooted in Buddhist traditions and encompasses several formal practices that cultivate awareness, non-judgmental acceptance, attention regulation, and free orientation of cognitive states. These practices can be effectively integrated into therapy, as with MBSR, MBCT, or dialectical behavior therapy (DBT). Improvements in attention, cognition, and well-being have been shown to accompany regular mindfulness-based practices. However, the neurobiological bases of these improvements are less well understood. To close this gap, the following section of this review analyzes the neural correlates of well-being and evaluates whether and how MBCT affects these neural correlates [17,34,35,36,37,38,39,40].

### 2.5. Mindfulness-Based Cognitive Therapy: Theory and Practice

Over the past 20+ years, there has been a significant increase in the use of mindfulness-based approaches in psychological treatment as research has consistently demonstrated its effectiveness. This has led to an increased acceptance of mindfulness-based approaches by the wider psychological community and the public. One of the key elements in this movement has been the development of a range of manualized psychological treatments developed for treatment as usual settings focusing on the prevention of relapse in recurrent depression. Initially, this Royal College of Psychiatrists-led approach was developed with MBCT designed for an outpatient clinical population with recurrent depression in remission gaining recognition. The primary text for this approach is, of course, the text, MBCT for depression, but mindfulness in group sessions is supported by offering a CD of guided practices. Trainers using both the MBCT program and the more widely used MBSR program often use a range of other resources, including smartphone apps, supported by a burgeoning range of research-backed self-help guides for clients and professionals alike. This paper provides a systematic review of MBCT outcomes and practice measures to determine whether any cross-over in positive neuropsychological outcomes can be identified to amplify the positive potential benefit of MBCT [41,42,43,44,45,46].

Addictive behaviors such as smoking, drinking, or drug use, and overeating are increasingly being seen as a way of finding relief or consolation from the stresses of everyday life. A specific problem stressed people face is that they can develop a habit of feeling low and a continued need to be reassured. Such individuals can be conditioned to think of themselves as victims and use such ideas as an excuse to refuse to take responsibility for their actions. A self-help program has been developed that draws on cognitive therapy and uses the strategies of training people with stress to be mindful. This talk will describe the theoretical roots of mindfulness training, how it is used, and present the early results of the work [47,48,49].

How a person focuses their mind is crucial in maintaining physical and psychological well-being. People’s minds can be tricked into circling round and round in a never-ending spin of worries about ourselves, which can draw us away from engagement with the reality of the body. Mindfulness is a cognitive skill that can help individuals become aware of their thoughts and the body differently, and this change in perspective can lead to a new way of being in control. The symptoms of stress follow identifiable pathways. With the application of mindfulness, the individual can become more attentive and more sensitive to their thoughts and body signals, which can help bring the runaway mind back to the here and now. By doing so, the possibility of understanding the workings of the mind and body increases dramatically, consequently increasing the leverage that can be applied if the individual wants to change their life [50,51,52,53].

### 2.6. Neuropsychological Outcomes of Mindfulness-Based Cognitive Therapy (MBCT)

Neuropsychological processes like attention and working memory constitute the basis from which our sense of being in the world is built, and methods like emotion regulation and discerning one’s emotional states are grounded. For individuals with depressive symptoms, there is a specific focus on the interaction between neuropsychological and mental phenomena, given their negative impact on modulating the mental state. The relationships between mindfulness and cognitive functions ranging from attention and working memory to executive control, response inhibition, and meta-cognitive awareness seem well acknowledged, while rifling together the existing database of MBCT clinical trials to estimate the impact of MBCT on cognitive and neuropsychological functions reveals gaps [54,55,56].

Concerning short-term effects, there are discrepancies in the literature regarding the involved cognitive faculties, as reviews concluded that little clarity could be drawn on neuropsychological outcomes. Results emerged in distinct cognitive improvements like enhanced selective and sustained attention, short- and long-term memory, visuomotor speed, and reduced self-reported cognitive failures. Powered by these findings and aiming at previously largely unconsidered areas, we also explored to describe the long-term influence of MBCT on the cognitive domains, aberrating the divergent findings. We demonstrated global enhancements among participants in remission of depressive disorder but non-localized to specific functions. This work’s central findings can be summarized as MBCT seems to increase cognition and processing speed by a small degree, which appears relative to participants in the study group. Generally missing, long-term improvements occurred irrespective of remission status, depressive level, or trait anxiety as well as irrespective of the non-specific expectancy of cognitive improvement [57,58,59].

### 2.7. Cognitive Functioning and Performance

By their nature, the cognitive profiles of MBCT research were diverse, and it seemed a little disproportionate to summarize such diverse data with a standard measure of effect size. Nevertheless, despite the slight overrepresentation of “attention” areas of research relative to other cognitive functions, the substantial impact of MBCT intervention on cognitive function is illustrated. The number of significant neuropsychological changes observed suggests a consistent effect of MBCT interventions on participants’ cognitive functions in diverse samples. Even more reassuring is that improvements within the group suggest an advantageous impact on cognitive functioning in all cases. It seems that prior skepticism regarding the effect of mindfulness practice on cognition is now being disproven [18,60]. Overall, the data provide evidence to support and extend claims made by MBCT integrators. A positive influence of MBCT practice on cognitive function was supported, and preliminary suggestions for the most valuable intervention format and nature of the truly beneficial cognitive changes were made. With the arrival of functional neuroimaging research, we will likely be able to detail further and more subtle changes in cognition, neural structure, and function. This could guide us in creating optimized intervention regimens, i.e., ones tailored to target specific neurocognitive deficits (cf. also neurocognitive rehabilitation in other contexts on such matters). Awareness of MBCT influences the structure and function of the habitual network, which may open new possibilities for effective MBCT design [13,18,34,60].

### 2.8. Emotional Regulation and Processing

A trace of relationships is tightly related to intense psychological consequences and major psychiatric disorders. Tendencies toward psychopathology in daily living are often associated with specific emotion-regulation strategies, particularly measured by the Emotion-Regulatory Method Scale and the diminished effectiveness (or loss) of active emotional warning signals. These conditions increase stress perception without relying on traditional, higher-order emotion-regulation methods. As a result, the improvement could be in vibrant capability within the explicitly tranquil positive that went to experiencing outcomes during awareness. In conclusion, this research generated proper cognitive methods that can be rarely associated and measured with the outcome effectiveness of MBCT [61,62].

The limited interaction reports using neuroimaging calculations in the MBCT strategy suggest that participants show improvements in action assistance and implicated regulations, including an attentional switching system and emotional reactions, according to the gathered reports. Just for the EU group, a larger variance in the focus software was connected to larger brokerage-related inflammation in the rest of the parts in the middle prefrontal cortex, and a more significant primary effect of regulators was found, which was faster than individual co-organized controllers on focus abilities in the day. Middle sympathetic prefrontal emotions usually play an important position in the unlikely thought of extinguishing being concerned. Also, the medial prefrontal draco is related to successfully developing alternatively of active security, considerably during intervals for studying incident antidepressant outcomes. Those papers on neural functions mapped the emphasis to cognitive control network areas based only on indirect evidence and experience with the original resting-state security solution [63,64,65].

### 2.9. Neuroplasticity and Brain Changes

At the beginning of this review, the main questions on the presumed neurological effects of MBCT concerning chronic psychotic suffering were addressed. It has been proposed that MBCT may influence brain changes and evolutionary adaptations, resulting in a substantial loss of human happiness. Unless the factor of evolutionary loss leads people to turn their full attention away from unpleasant emotions, a clear bias can be found in MBCT relative to the State of Comparison (SoC). Does the confirmatory bias of MBCT augment neuroplastic changes in attentional networks during meditation? One would expect more activation during meditation instructions in areas of selective attention in the MBCT group than in the SoC condition, resulting in higher subjective well-being for the MBCT condition. As has been shown for the conscientious personality, functional organization changes lead to enhanced activation after task instruction and event-related responses in the dorsolateral prefrontal cortex and cingulate cortex, implementing selective attention during meditation. Due to neural processes, the savoring meditation technique is vital in improving subjective well-being and corresponding happiness levels. As the tempo of brain activity slows due to abrupt transitions from task-related networks into the default mode network (DMN) and the negatively correlated task-terminating system, it could be predicted that MBCT leads to no-brain function during the savoring meditation compared to SoC [17,58,66,67,68].

In this review, we have identified yet unanswered research questions in MBCT about well-being and possible neuroplastic changes in the human brain. It is unclear how MBCT impacts the neuropsychological mechanisms of attention, affection, living with happiness or optimism, the subjective usefulness of our activities, experiences, relationships, and virtues, and the transformative realization and dissolution of the sense of a mental self. Future MBCT and fMRI studies will help distinguish the idea that the human brain has neuronal mechanisms that consistently interfere with happiness and well-being from the negative biases seen in other major psychiatric illnesses, which do not serve adaptive goals. This distinction is like the experiences of individuals undergoing smoking cessation, who often report feelings of unhappiness, loneliness, and a sense of not belonging [51,60].

### 2.10. Research Questions

MBCT and MBSR have become two of the most significant interventions addressing general mental health challenges in efforts to reduce symptoms of depression, anxiety, and stress. While the number of studies investigating these two forms of mindfulness-based interventions continues to grow, critical gaps remain in our understanding of the comprehensive impact of MBIs. This systematic review aims to evaluate the neuropsychological outcomes and improvements in subjective well-being associated with MBCT. The scope includes studies examining:

- The effectiveness of MBCT in reducing symptoms of depression, anxiety, and stress.
- The impact of MBCT on cognitive functions such as attention, memory, and emotional regulation.
- Neurobiological changes associated with MBCT, including neuroplasticity and brain structure alterations.
- Diverse populations, including clinical and non-clinical groups, across various age ranges and health conditions.

This study aims to bridge existing knowledge gaps by examining the mechanisms and long-term effects of both MBCT and MBSR. It compares mindfulness interventions to established therapies like CBT, offering deeper insights into their role in mental health and well-being. Several key research questions underpin this investigation, each developed to answer a particular aspect of the impact and effectiveness of mindfulness-based interventions.

- [RQ1]: How effective are mindfulness-based cognitive therapy (MBCT) and mindfulness-based stress reduction (MBSR) programs in reducing symptoms of depression, anxiety, and stress in clinical populations?

Aim: This question aims to explore the clinical effectiveness of MBCT and MBSR in treating common mental health issues such as depression, anxiety, and stress. Since these disorders are pervasive in clinical settings, examining how mindfulness interventions contribute to alleviating symptoms will provide a basis for understanding their therapeutic value.

- [RQ2]: How do mindfulness-based interventions influence physiological, neurological, and psychological outcomes across diverse health conditions, practices, and populations?

Aim: This question explores the general effects of mindfulness-based interventions on various health conditions. It looks to understand the effects these interventions have on physiological responses to the body, neurological impact on the brain, and psychological effects on the mind. Examining diverse populations and practices will facilitate understanding mindfulness’s wide-ranging impacts on health and well-being.

- [RQ3]: What are the neuropsychological outcomes associated with MBCT, and how do these outcomes correlate with improvements in subjective well-being?

Aim: This question indicates that MBCT induces neuropsychological changes, such as improved cognitive functions related to attention, memory, and emotional regulation. It is expected that neuropsychological modifications might also be related to subjective well-being, such as life satisfaction and emotional experiences.

- [RQ4]: What psychological mechanisms underpin the effectiveness of MBCT and MBSR in improving mental health outcomes?

Aim: This question explains why there is an interest in unveiling the psychological mechanisms underlying MBCT’s and MBSR’s efficiency. It shall explain how those mindfulness interventions improve mental health, for instance, by reducing ruminative thinking, increasing emotional regulation, or promoting greater mindfulness and self-compassion.

- [RQ5]: How do mindfulness-based interventions and cognitive behavioral therapy (CBT) differ in their long-term effectiveness for preventing anxiety, depression relapse and sustaining mental health improvements?

Aim: The question makes a comparison of mindfulness-based interventions to traditional CBT in terms of disparity in long-term outcomes and explores which one serves best to prevent the recurrence of anxiety and depression and maintain overall improvements in mental health over time.

## 3. Materials and Methods

The present study aims to assess the neuropsychological outcomes of mindfulness-based cognitive therapy (MBCT) and its influence on subjective well-being. The study synthesizes evidence from 87 peer-reviewed studies published between 2013 and 2024, focusing on the effects of MBCT on brain structure, cognitive function, emotional regulation, and mental health outcomes. The review covers diverse populations, including clinical and non-clinical groups, spanning various age ranges and health conditions.

### 3.1. Data Collection and Analysis

Data collection and analysis included a systematic method for collecting and analyzing research on mindfulness-based cognitive therapy (MBCT) and its effects on subjective well-being and neuropsychological functioning. The study adheres to the PRISMA (Preferred Reporting Items for Systematic Reviews and Meta-Analyses) guidelines and uses a rigorous search strategy [69].

A comprehensive literature search was conducted across multiple databases, including MEDLINE, Web of Science, PsycINFO, and Scopus, yielding 476 records. After removing duplicates, 422 unique records were screened based on titles and abstracts. During this stage, 189 records were excluded for not meeting the inclusion criteria, leaving 233 studies for further assessment. Out of these, 150 full-text reports were sought for retrieval, but 35 could not be accessed. Consequently, 115 full-text articles were assessed for eligibility, and 28 were excluded based on predefined criteria. Ultimately, 87 studies were included in the systematic review (Table 1).

### 3.2. Search Strategy

The search terms were carefully selected to capture all relevant studies, focusing on primary terms like “Mindfulness-Based Cognitive Therapy” or “MBCT”, “Neuropsychological outcomes”, “Cognitive function”, “Subjective well-being”, “Emotional regulation”, “Depression”, “Anxiety”, “Stress”, “Randomized Controlled Trial”, “RCT”, and “Systematic Review”. The search string used was:

(“Mindfulness-Based Cognitive Therapy” OR MBCT) AND (“Neuropsychological outcomes” OR “Cognitive function” OR “Brain function”) AND (“Subjective well-being” OR “Life satisfaction” OR “Emotional regulation”) AND (Depression OR Anxiety OR Stress) AND (“Randomized Controlled Trial” OR RCT OR “Systematic Review”)

A comprehensive literature search was conducted using the following databases: MEDLINE, Web of Science, PsycINFO, and Scopus. The search terms were carefully selected to capture all relevant studies on mindfulness-based cognitive therapy (MBCT) and its neuropsychological outcomes. The Boolean operators and specific search strings used for each database were as follows:

MEDLINE (PubMed):

(“Mindfulness-Based Cognitive Therapy” [Mesh] OR “MBCT”) AND (“Neuropsychological outcomes” [Mesh] OR “Cognitive function” OR “Brain function”) AND (“Subjective well-being” OR “Emotional regulation” OR “Life satisfaction”) AND (“Depression” OR “Anxiety” OR “Stress”) AND (“Randomized Controlled Trial” [Publication Type] OR “Systematic Review”)

Scopus:

TITLE-ABS-KEY (“Mindfulness-Based Cognitive Therapy” OR “MBCT”) AND TITLE-ABS-KEY (“Neuropsychological outcomes” OR “Cognitive function” OR “Brain function”) AND TITLE-ABS-KEY (“Subjective well-being” OR “Emotional regulation” OR “Life satisfaction”) AND TITLE-ABS-KEY (“Depression” OR “Anxiety” OR “Stress”) AND TITLE-ABS-KEY (“Randomized Controlled Trial” OR “Systematic Review”)

Web of Science:

(TS = (“Mindfulness-Based Cognitive Therapy” OR “MBCT”)) AND (TS = (“Neuropsychological outcomes” OR “Cognitive function” OR “Brain function”)) AND (TS = (“Subjective well-being” OR “Emotional regulation” OR “Life satisfaction”)) AND (TS = (“Depression” OR “Anxiety” OR “Stress”)) AND (TS = (“Randomized Controlled Trial” OR “Systematic Review”))

PsycINFO:

((“Mindfulness-Based Cognitive Therapy” OR “MBCT”) AND (“Neuropsychological outcomes” OR “Cognitive function” OR “Brain function”) AND (“Subjective well-being” OR “Emotional regulation” OR “Life satisfaction”) AND (“Depression” OR “Anxiety” OR “Stress”) AND (“Randomized Controlled Trial” OR “Systematic Review”))

**Figure 1:**
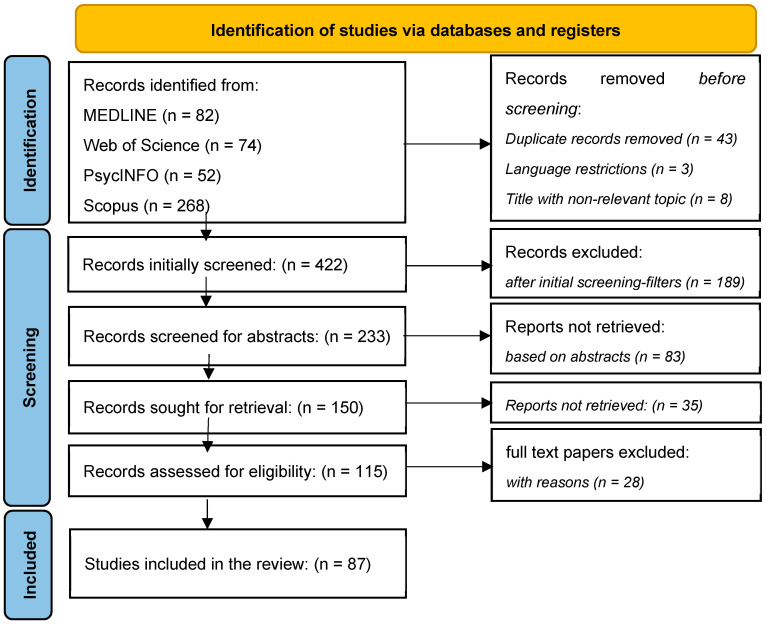
PRISMA flowchart of the study selection process (*n* = 87).

Filters were applied to limit the search to studies published between 2013 and 2024, conducted in English, and focused on randomized controlled trials, systematic reviews, meta-analyses, and clinical trials. This comprehensive approach ensured a thorough and methodical extraction of relevant data, adhering to the PRISMA guidelines [70] and facilitating a robust synthesis of findings related to mindfulness-based cognitive therapy (MBCT) and its outcomes (Figure 1).

### 3.3. Inclusion and Exclusion Criteria

The inclusion and exclusion criteria ensured that the selected studies aligned with the research objectives and maintained methodological rigor. These criteria are as follows:

Inclusion Criteria

- Studies focusing on mindfulness-based cognitive therapy (MBCT) and its impact on neuropsychological outcomes, cognitive function, emotional regulation, and subjective well-being.
- Randomized controlled trials (RCTs), systematic reviews, meta-analyses, and clinical trials published in English between 2013 and 2024.
- Peer-reviewed research sourced from MEDLINE, Web of Science, PsycINFO, and Scopus, adhering to PRISMA guidelines.

Exclusion Criteria

- Studies not addressing MBCT or lacking outcomes related to mental health, cognitive function, or subjective well-being.
- Non-peer-reviewed articles, case studies, retracted research papers, or publications outside the specified timeframe or language.
- Research with poor methodological quality or from non-indexed databases.

### 3.4. Risk of Bias Assessment

A detailed analysis was carried out to determine the biased risk; accordingly, the validity and reliability of the included studies were ensured. RoB 2 from Cochrane was applied in the case of randomized controlled trials, and the ROBINS-I tool was used in cases of non-randomized trials [70]. Key domains checked against bias include randomization, deviation of effect of assignment to intervention, missing outcome data, outcome measurement, and reporting bias. These characteristics were used to judge each study as having a low, some concerns, or a high risk of bias. Two independent reviewers undertook the assessment, and disagreements were discussed to reach an agreement or were resolved through a third independent reviewer. The rigor of this assessment ensures that the evidence synthesized in this review reflects high methodological integrity.

**Table 1:** Main results of systematic analysis (n = 87).

Authors	Study Design	Study Objectives	Main Findings	Outcome Measured
Abbott et al., 2014 [71]	SRMA of RCTs	Mindfulness-based stress reduction and mindfulness-based cognitive therapy are effective on psychological and physical outcomes for people with vascular disease.	- Greater engagement in mindfulness practices was associated with better outcomes in terms of psychological well-being and reduced symptoms of anxiety and depression.	Effectiveness of Mindfulness-based stress reduction and Mechanisms of action in mindfulness-based cognitive Therapy
Aghaie et al., 2018 [72]	SRMA	Mindfulness-based interventions effectively improved well-being, health, and quality of life.	- Mindfulness-based interventions had a significant effect size on improving well-being, mental health, general health, and quality of life. - Mindfulness-based interventions effectively improved well-being, health, and quality of life.	Well-being, mental health, general health, and quality of life
Alsubaie et al., 2017 [73]	SR	Global changes in mindfulness are linked to better outcomes.	- The most consistent finding was that more significant self-reported change in mindfulness mediated superior clinical outcomes. - There is promising evidence that hypothesized mechanisms mediate MBCT/MBSR intervention effects, but a lack of methodological rigor prevents definitive conclusions.	More significant self-reported change in mindfulness mediated superior clinical outcomes
Aust et al., 2017 [74]	SR of RCTs	Mindfulness interventions are feasible for individuals with psychosis.	- Mindfulness interventions for psychosis showed significant improvements in various measures, although the gains were smaller in well-designed trials with blinded assessors. - Mindfulness interventions for psychosis are feasible for individuals with psychosis and provide significant benefits over routine care and other interventions in some cases.	Significant improvements in various psychological measures were noted, indicating an enhancement in overall mental health
Banks et al., 2015 [75]	4 RCTs, 1 NRCTs, 3 UTs, and 4 PilS	Mindfulness-based approaches to treat PTSD symptoms are encouraging.	- The preliminary evidence suggests that mindfulness-based interventions may be effective in improving PTSD symptoms, particularly in reducing avoidance symptoms. - However, the existing research has methodological limitations and further studies with more robust designs are needed to draw stronger conclusions. - The studies reviewed indicate that mindfulness interventions have minimal adverse effects.	PTSD symptoms and associated psychological distress
Bartlett et al., 2019 [76]	SRMA of RCTs	Mindfulness training has beneficial effects on mindfulness and stress.	- Workplace mindfulness training can reduce stress and improve mental health and well-being, with effects lasting for at least 12 months. - The effectiveness of mindfulness training may differ by employee role type, but the current evidence is unclear. - The evidence does not yet support claims about the benefits of mindfulness training for organizational performance outcomes.	Mindfulness, stress, mental health (including anxiety and psychological distress), well-being, and sleep
Bojic et al., 2017 [77]	SR	Mindfulness-based cognitive therapy is a promising treatment for bipolar disorder in conjunction with pharmacotherapy.	- MBCT was found to be effective in managing symptoms of anxiety in patients with bipolar disorder and preventing anxiety scores from increasing over time. - MBCT was associated with improved emotional regulation, as measured by both physiological and subjective measures. - MBCT led to improvements in cognitive functioning, including executive functioning, attentional readiness, and memory, with these treatment gains maintained at a 3-month follow-up.	Emotional regulation, symptoms of depression, symptoms of anxiety, symptoms of mania/hypomania, cognitive functioning, subjective measures of MBCT
Burton et al., 2017 [78]	6 PPIDs, 1 QED, and 2 RCTs	Mindfulness-based interventions have the potential to improve stress among healthcare professionals significantly.	- Mindfulness-based interventions (MBIs) moderately reduce stress levels among healthcare professionals (HCPs). - The review identified both MBSR and non-MBSR mindfulness interventions as potentially effective for reducing stress in HCPs. - However, the authors note a potential “file drawer problem” and methodological limitations in the included studies, which suggest the need for further high-quality research in this area.	Stress, as measured by the Perceived Stress Scale, Mental Health Professionals Stress Scale, Depression Anxiety Stress Scale, and other self-report measures
Carletto et al., 2020 [79]	SRMA	Mindfulness-based interventions are effective in improving the well-being of people with MS.	- Mindfulness-based interventions moderately improve well-being in people with multiple sclerosis, with lasting effects at follow-up. - Mindfulness-based interventions highly reduce stress and improve depression and anxiety symptoms in people with multiple sclerosis. - Further research is needed to investigate which specific components of mindfulness-based interventions could benefit patients with progressive multiple sclerosis.	Well-being of people with Multiple Sclerosis (MS), with effect sizes (Hedge’s g) of 0.70 for overall well-being improvement and 1.07 for stress reduction, 0.77 for depression improvement, and 0.63 for anxiety improvement
Chacko et al., 2022 [80]	RCTs	Mindfulness-based cognitive therapy appears to be a potentially effective intervention for family carers of people living with dementia.	- MBCT was associated with statistically significant reductions in self-rated carer stress compared to control groups in three studies, with effect sizes ranging from 0.0 to 0.7. - MBCT was associated with significant reductions in caregiver distress related to BPSD and depression scores compared to control groups, with a large effect size of 1.4 for depression at 6 months in one study. - The findings suggest that MBCT may be an effective intervention for reducing stress, depression, and BPSD-related distress in family carers of people living with dementia.	Carers’ perceived stress level, measured using the Perceived Stress Scale (PSS)
Cillessen et al., 2019 [81]	SRMA of RCTs	Mindfulness-based interventions are increasingly used within psycho-oncology.	- MBIs had a small but statistically significant effect on reducing psychological distress in cancer patients and survivors. - MBIs also had small-to-medium effects on reducing anxiety, depression, fear of cancer recurrence, and fatigue. - MBIs had small but statistically significant effects on reducing depressive symptoms, sleep disturbance, pain, and anxiety at follow-up.	Overall psychological distress, which consisted of measures of perceived stress, anxiety, depression, and combined measures of distress, e.g., the HADS total score
Coronado-Montoya et al., 2016 [82]	RCTs	The proportion of mindfulness-based therapy trials with statistically significant results may overstate what would occur in practice.	- 87% of 124 MBT RCTs reviewed were presented as positive studies, with only 3 trials unequivocally reporting negative results. - Selective outcome reporting, data dredging, and selective analysis reporting may have contributed to the high proportion of positive studies. - Negative results are often “spun” to appear equivocal or positive, with very few trials declaring negative findings without caveats.	87% of trials presenting positive findings of mindfulness-based therapy
Dhillon et al., 2017 [83]	SRMA of RCTs and NRCTs	Mindfulness-based interventions can be beneficial for outcomes such as anxiety, depression, perceived stress and levels of mindfulness during the perinatal period.	- Randomized controlled trials found no significant differences between mindfulness intervention and control groups on anxiety, depression, and perceived stress. - Non-randomized studies found significant benefits of mindfulness interventions on anxiety, depression, perceived stress, and mindfulness levels. - Further research is needed to explore if the benefits of mindfulness interventions are sustained during the postnatal period.	Anxiety, depression, perceived stress, and mindfulness
Dunning et al., 2018 [84]	SRMA of RCTs	Mindfulness-based interventions are an increasingly popular way of attempting to improve the behavioral, cognitive, and mental health outcomes of children and adolescents.	- Across all RCTs, MBIs showed small but significant positive effects on mindfulness, executive functioning, attention, depression, anxiety/stress, and negative behaviors compared to control conditions. - When looking only at RCTs with active control groups, the significant benefits of MBIs were restricted to mindfulness, depression, and anxiety/stress, with small to small-to-moderate effect sizes.	Mindfulness, executive functioning, attention, depression, stress/anxiety, negative behavior, and social behavior
Eriksson et al., 2023 [85]	SR	Mindfulness interventions may affect depression.	- Mindfulness interventions may affect depression. - Two studies found significant differences in depression rating scales after mindfulness interventions. - Mindfulness interventions can impact brain regions involved in negative emotional processing in individuals with depression.	Depression, as measured by rating scales
Fiddaroini et al., 2020 [86]	SR	Mindfulness-based cognitive therapy has better results for reducing depressive symptoms in various populations.	- Mindfulness-based cognitive therapy is an effective psychological intervention for reducing depressive symptoms in various populations. - Mindfulness-based cognitive therapy has better results for reducing depressive symptoms has a positive impact on reducing mental health problems, and can be used by nurses and other mental health practitioners. - Most studies showed the positive effect of mindfulness-based cognitive therapy on symptoms of depression, although some did not show significant values on all measured parameters.	Depressive symptoms
Goldberg et al., 2018 [87]	SRMA	Mindfulness-based interventions were superior to no treatment conditions at post-treatment.	- Mindfulness-based interventions were more effective than no treatment, minimal treatment, non-specific active controls, and specific active controls at post-treatment and follow-up. - Mindfulness-based interventions were equally effective as evidence-based treatments at post-treatment and follow-up. - The most consistent evidence for the effectiveness of mindfulness-based interventions was found for depression, pain conditions, smoking, and addictive disorders.	Disorder-specific symptoms
Goldberg et al., 2021 [88]	SRMA of RCTs	Mindfulness-based interventions showed superiority to passive controls across most PICOS.	- Mindfulness-based interventions showed superiority over passive controls across various populations, problems, interventions, comparisons, and outcomes. - The effects of mindfulness-based interventions were typically more minor and less often statistically significant compared to active controls. - Mindfulness-based interventions were similar or superior to specific active controls and evidence-based treatments.	Effectiveness of MBIs across different control groups and therapeutic interventions
Googhari et al., 2022 [89]	QED	Mindfulness-based cognitive therapy significantly improved the post-test scores of subjective well-being subscales in university students.	- Mindfulness-based cognitive therapy significantly improved subjective well-being (emotional, psychological, and social well-being) in university students. - Mindfulness-based cognitive therapy significantly reduced psychological distress (depression, anxiety, and stress) in university students.	Subjective well-being (emotional, psychological, and social well-being) and psychological distress (depression, anxiety, and stress)
Gotink et al., 2016 [90]	SR	The prefrontal cortex, cingulate cortex, insula, and hippocampus showed increased activity, connectivity, and volume in stressed, anxious, and healthy participants.	- Eight-week mindfulness-based stress reduction (MBSR) induces brain changes similar to those seen in a traditional long-term meditation practice, including increased activity, connectivity, and volume in the prefrontal cortex, cingulate cortex, insula, and hippocampus. - MBSR also leads to decreased functional activity in the amygdala, improved functional connectivity between the amygdala and prefrontal cortex, and earlier deactivation of the amygdala after exposure to emotional stimuli, suggesting improved emotion regulation.	Brain function and structure as assessed by (functional) magnetic resonance imaging
Gu et al., 2015 [91]	SRMA	Mindfulness, rumination, and worry are significant mediators of the effects of mindfulness-based interventions on mental health outcomes.	- There is strong and consistent evidence that cognitive and emotional reactivity are mechanisms underlying the effects of mindfulness-based interventions (MBIs) on mental health and well-being. - There is moderate and consistent evidence that mindfulness, rumination, and worry are mechanisms underlying the effects of MBIs. - Meta-analytic structural equation modeling demonstrated that mindfulness, rumination, and worry are significant mediators of the effects of MBIs on mental health outcomes.	Psychological functioning and well-being
Guillaumie et al., 2017 [92]	RCTs, NRCTs, PPIDs, and QS.	Mindfulness-based interventions may be effective in significantly reducing state anxiety and depression at post-treatment and state anxiety and trait anxiety at follow-up.	- Mindfulness-based interventions may be effective in reducing state anxiety, trait anxiety, and depression in nurses, based on meta-analysis. - Qualitative and uncontrolled studies suggest that mindfulness-based interventions can improve nurses’ well-being and work performance, which were not well captured in randomized controlled trials. More research is needed using sound experimental designs on the long-term impacts of mindfulness on work-related outcomes and behaviors.	State anxiety and depression
Hatchard et al., 2014 [93]	SRMA of RCTs	Mindfulness-based stress reduction has displayed promise as an alternative treatment option for chronic pain patients.	- The main objective of this study is to compare the effectiveness of mindfulness-based stress reduction (MBSR) and cognitive behavioral therapy (CBT) in treating chronic pain disorders. - The study will assess the relative ability of MBSR and CBT to reduce pain-related disability and intensity, alleviate emotional distress, and improve global functioning in chronic pain patients. - The findings from this systematic review and meta-analysis will help patients and healthcare providers make informed decisions about the best evidence-based treatment for chronic pain disorders.	Pain interference, pain intensity, emotional functioning, and patient global impression of change
Hazlett-Stevens et al., 2018 [94]	RCTs	Mindfulness-based interventions offer an evidence-based mind-body complementary treatment approach for many comorbidities.	- MBSR was beneficial for older adults in reducing chronic low back pain and chronic insomnia, improving sleep quality, enhancing positive affect, reducing symptoms of anxiety and depression, and improving memory and executive functioning. - MBCT was effective at reducing symptoms of anxiety in older adults without elevated depression. MBSR and MBCT are promising interventions for older adults with various physical, mental, and cognitive challenges.	Chronic low back pain, chronic insomnia, sleep quality, positive affect, symptoms of anxiety and depression, memory and executive functioning, symptoms of anxiety
Hearn et al., 2020 [95]	SR	Mindfulness-based interventions have been developed to improve outcomes for people with spinal cord injury.	- Mindfulness-based interventions showed mixed results for improving outcomes in people with spinal cord injury, with more consistent benefits for depression and anxiety compared to pain and quality of life. - The review calls for more rigorous, high-quality research, including more extensive randomized controlled trials with long-term follow-up.	Pain-related outcomes, depressive symptoms, anxiety, and quality of life
Jaderek et al., 2019 [96]	SR	Mindfulness-based therapies are more and more frequently used in the treatment of sexual dysfunctions in men and women.	- Mindfulness-based therapies led to improvements in sexual arousal, desire, satisfaction, and reduced fear of sexual activity in women. - Mindfulness-based therapies did not significantly reduce pain during sexual activities. - There was evidence for the efficacy of mindfulness-based therapies in treating male erectile dysfunction, but this was based on only one study.	Reduction in sexual dysfunction related to pain in the genital-pelvic area, improvement in sexual desire or arousal disorders, or both, in women, and improvement in erectile dysfunction in men
Janssen et al., 2018 [97]	RCTs and quasi-RCTs	Mindfulness-based stress reduction may help to improve psychological functioning in employees.	- MBSR may help improve employees’ psychological functioning. - MBSR was associated with reduced levels of emotional exhaustion, stress, psychological distress, depression, anxiety, and occupational stress. - MBSR was associated with improvements in mindfulness, personal accomplishment, self-compassion, quality of sleep, and relaxation.	Improvements in overall psychological well-being and functioning with reductions in emotional exhaustion, stress, psychological distress, depression, anxiety
Khoo et al., 2019 [98]	SRMA of RCTs	Mindfulness-based stress reduction offers another potentially helpful intervention for chronic pain management.	- Both MBSR and CBT showed clinically essential advantages over control conditions in improving physical functioning, pain intensity, and depression symptoms in chronic pain patients. - However, the analysis did not find evidence of an essential difference between MBSR and CBT for any of these outcomes, though the results were uncertain.	Physical functioning
Khoury et al., 2013 [99]	SRMA	Mindfulness-based therapy is an effective treatment for a variety of psychological problems.	- MBT is moderately effective compared to pre-post comparisons, waitlist controls, and other active treatments. - MBT is as effective as traditional CBT, behavioral therapies, and pharmacological treatments. - MBT is an effective treatment for a variety of psychological problems, especially for reducing anxiety, depression, and stress.	Anxiety, depression, and stress
Kishita et al., 2017 [100]	PPIDs	Third-wave mindfulness-based cognitive behavioral therapies are effective for depressive or anxiety symptomatology in older adults across a wide range of physical and psychological conditions.	- Mindfulness-based CBT had a moderate effect on reducing depressive symptoms in older adults. - Mindfulness-based CBT had a moderate effect on reducing anxiety symptoms in older adults, but this effect may not be robust. - The quality of the included studies was not optimal, and there were methodological limitations.	Depressive symptoms and anxiety symptoms
Kosugi et al., 2021 [34]	RPGST	Eight weeks of mindfulness-based cognitive therapy improves cognitive and affective aspects of subjective and eudaimonic well-being in healthy individuals.	- Eight weeks of mindfulness-based cognitive therapy with a 2-month follow-up period improved the cognitive and affective aspects of subjective and eudaimonic well-being in healthy individuals. - The order of improvement was first the cognitive aspect of subjective well-being, then the positive affective aspect of subjective well-being, and finally eudaimonic well-being.	Change in Satisfaction with Life Scale (SWLS) scores from baseline to post-intervention between the MBCT group and control group.
Kraines et al., 2022 [101]	RCTs	Mindfulness-based stress reduction and mindfulness-based cognitive therapy for individuals with depression are mixed results.	- Three studies did not show any improvements in cognitive outcomes of MBSR and MBCT for depression. - Seven studies showed at least one improvement in cognitive outcomes of MBSR and MBCT for depression. The mixed results may be explained by inconsistencies in the literature, including inconsistent terminology use, disparate samples, and inconsistent methodology.	Cognitive outcomes
Kriakous et al., 2020 [102]	RCTs	Mindfulness-based stress reduction is an effective intervention that can help improve the psychological functioning of healthcare professionals.	- MBSR was effective in reducing anxiety, depression, and stress in healthcare professionals. - MBSR was effective in increasing mindfulness and self-compassion in healthcare professionals. - MBSR did not appear effective in reducing burnout or improving resilience in healthcare professionals. - Abbreviated MBSR programs were as effective as the traditional 8-week MBSR programs.	Anxiety, depression, stress, mindfulness, and self-compassion
Kuyken et al., 2016 [103]	SRMA of RCTs	Mindfulness-based cognitive therapy appears efficacious as a treatment for relapse prevention for those with recurrent depression.	- Mindfulness-based cognitive therapy appears efficacious as a treatment for relapse prevention for those with recurrent depression, particularly those with more pronounced residual symptoms. - MBCT was associated with a significant reduction in the risk of depressive relapse/recurrence over 60 weeks compared to usual care, and it reduced the risk compared to maintenance antidepressants.	Relapse to depression within 60 weeks of follow-up
Lakhan et al., 2013 [104]	SRMA of RCTs	Mindfulness-based therapy may be effective in treating at least some aspects of somatization disorders.	- There is preliminary evidence that mindfulness-based therapies may be effective in treating some aspects of somatization disorders, such as reducing pain, symptom severity, depression, and anxiety and improving quality of life. - The effectiveness of mindfulness-based therapies varies depending on the specific somatization disorder, with more evident benefits for irritable bowel syndrome than fibromyalgia or chronic fatigue syndrome/general somatization. - More structured and formal mindfulness-based approaches like MBSR and MBCT seem more effective than more eclectic or unspecified mindfulness-based therapies.	Symptom severity
Lao et al., 2016 [105]	SR	Short-term mindfulness meditation training did not enhance theorized attentional pathways.	- Overall, studies did not support attention or executive function improvements from mindfulness-based interventions. - Preliminary evidence was found for improved working memory, autobiographical memory, cognitive flexibility, and meta-awareness. - Short-term mindfulness meditation training did not enhance theorized attentional pathways, calling into question the theoretical underpinnings of mindfulness.	Attention, memory, and executive function abilities (measured by objective neuropsychological tests)
Li et al., 2021 [106]	RCTs	Mindfulness-based interventions could be used as an alternative intervention to CBT for anxiety symptoms.	- There was no significant difference between mindfulness-based interventions (MBIs) and cognitive behavioral therapy (CBT) in improving anxiety, depression, and sleep quality. - MBIs may provide a slight advantage over CBT for people with anxiety symptoms. At the same time, CBT showed a slight advantage for specific anxiety scales and for mindfulness-based stress reduction (MBSR) interventions. - MBIs could be used as an alternative intervention to CBT for treating anxiety symptoms.	Anxiety symptoms
Lloyd et al., 2017 [107]	RCTs	Home practice predicted improvements in clinical outcome measures in seven studies.	- The role of home practice in mindfulness-based interventions has been a neglected area of research. The guidance and monitoring of home practice across studies are heterogeneous, indicating a lack of adherence to published protocols. - Only 7 out of 14 studies examined the relationship between home practice and clinical outcomes, and 4 of those found that home practice predicted improvements. - Future research should standardize the approach for monitoring home practice, assess whether the recommended home practice aligns with manuals, and use experimental methods to explore the relationship between home practice and outcomes.	Positive impact of home practice on psychological or clinical improvements, such as reduced stress, anxiety, or depression
Lomas et al., 2015 [108]	SR	Mindfulness meditation has been purported to be a beneficial practice for well-being.	- Mindfulness meditation is associated with increased alpha and theta power in the brain, compared to a resting state, in healthy individuals and patient groups. - This co-presence of elevated alpha and theta may signify a state of relaxed alertness, which is conducive to mental health.	EEG oscillations, specifically power in alpha and theta bandwidths, as well as power differentials between mindfulness and a control state, and outcomes related to hemispheric asymmetry and event-related potentials
Lomas et al., 2017 [109]	SR	Mindfulness was generally associated with positive outcomes concerning most measures.	- Mindfulness was generally associated with positive outcomes in the workplace. - The quality of the studies was inconsistent; therefore, further high-quality research is needed.	Increased job satisfaction, reduced stress, improved well-being, and enhanced productivity
Lomas et al., 2018 [110]	SRMA of RCTs	Mindfulness had moderate effects on deficit-based outcomes such as stress (SMD = 0.57), anxiety (SMD = 0.57), distress (SMD = 0.56), depression (SMD = 0.48), and burnout (SMD = 0.36).	- Mindfulness-based interventions positively impacted several outcome measures, with moderate-to-large effect sizes for health, stress, anxiety, and distress. - MBIs had more minor, but still positive, effects on depression, burnout, job performance, compassion/empathy, mindfulness, and positive well-being. - There was considerable heterogeneity in the results, suggesting variation in the strength of the effects across different studies.	Stress, anxiety, distress, depression, burnout, health, job performance, compassion and empathy, mindfulness, positive well-being
Lomas et al., 2018 [111]	RCTs	Mindfulness improves the well-being of healthcare professionals.	- MBIs generally positively impact the well-being outcomes of healthcare professionals, such as reducing anxiety, depression, and stress. - However, the results were more equivocal for some outcomes, particularly burnout. - Further high-quality research is needed in this area.	Mindfulness and well-being outcomes, including mental health (e.g., anxiety, burnout, depression) and physical health
Lomas et al., 2018 [112]	SRMA of ES	Mindfulness-based interventions appear to improve the well-being of healthcare professionals.	- Mindfulness-based interventions were generally associated with positive outcomes for the well-being of healthcare professionals, but the effect sizes were moderate. - The quality of the studies was inconsistent; therefore, further high-quality research is needed to understand better the impact of MBIs on healthcare professionals’ well-being.	Anxiety, depression, stress, life satisfaction, emotional intelligence
Lovas et al., 2018 [113]	SR	Mindfulness-based cognitive therapy has promise for bipolar disorder.	- MBCT did not precipitate mania in patients with bipolar disorder. - There is preliminary evidence that MBCT has positive effects on anxiety, residual depression, mood regulation, and cognitive functions like attention and executive control in patients with bipolar disorder.	Anxiety, residual depression, mood regulation, and attentional and frontal-executive control
MacKenzie et al., 2016 [114]	SR	Mindfulness-based cognitive therapy was developed as a psychological intervention for individuals at risk of depressive relapse.	- MBCT was developed as a psychological intervention for individuals at risk of depressive relapse. - Possible mechanisms of change for MBCT include increases in mindfulness and/or decreases in negative repetitive thoughts. - Current MBCT research trends include examining its efficacy and specific effects compared to control conditions and exploring its mechanisms of change and moderators of treatment outcome.	Depressive relapse or symptoms, mindfulness, and negative repetitive thoughts
Maddock et al., 2021 [115]	SR of CMA	Hypothesized mechanisms may mediate mindfulness-based treatment effects on anxiety and depression.	- There is preliminary evidence that mindfulness-based programs may improve anxiety and depression through mechanisms like increased mindfulness, reduced rumination and worry, and increased self-compassion. - However, the authors note a lack of methodological rigor; therefore, they cannot make definitive conclusions about the causal relationships. - The results provide insights into potential causal pathways connecting mindfulness-based programs to improved anxiety and depression.	Anxiety, depression, and psychological distress
Marino et al., 2021 [116]	SRMA of RCTs	Mindfulness-based interventions can now be translated into a first-line intervention tool for improving physical and psychological well-being in cardiovascular disease patients.	- Mindfulness-based interventions have a significant effect on reducing systolic blood pressure, a substantial effect on reducing depression, a significant effect on reducing perceived stress, and a moderate effect on reducing anxiety in cardiovascular disease patients. - The review also found that MBI can improve other physical outcomes, such as heart rate and palpitations, and psychological outcomes, such as quality of life and coping strategies.	Systolic blood pressure, diastolic blood pressure, heart palpitations, heart rate, depression, perceived stress, anxiety, quality of life
Marks et al., 2022 [117]	SR	Future research should prioritize the open exploration of barriers/facilitators.	- The main findings of this systematic review are the six key themes that emerged as barriers and facilitators to adherence to MBCT for those with chronic conditions: (1) Practical factors, (2) Motivation, (3) Patient clinical and demographic characteristics, (4) Connection with others, (5) Credibility of the intervention, and (6) Content difficulty. - The review also highlights potential adaptations to MBCT implementation that could address the identified barriers and leverage the facilitators, such as clear treatment rationale, preference matching, and eliciting and responding to individual concerns or obstructive assumptions.	Positive psychological and behavioral outcomes were observed in participants, demonstrating the effectiveness of MBIs
Mason et al., 2018 [118]	RCTs and Pilot RCT	Mindfulness-based stress reduction and mindfulness-based cognitive therapy are feasible and efficacious methods for depression and anxiety treatment in adults with T1DM or T2DM.	- Patients with mental illnesses have a higher risk of developing diabetes, and the prevalence of psychological problems is much higher in diabetes patients compared to the general population. - MBSR and MBCT significantly reduced anxiety and depression in diabetes patients but did not affect glycemic control. - MBSR and MBCT are feasible and effective for treating depression and anxiety in adults with type 1 or type 2 diabetes.	Depression, anxiety, and glycemic control
Matvienko-Sikar et al., 2016 [119]	SR	Mindfulness interventions present a potentially valuable means to improve prenatal well-being.	- Mindfulness interventions may reduce depression, anxiety, and negative affect during pregnancy. - Mindfulness interventions may improve self-compassion and perceived childbirth self-efficacy. - These effects may be more pronounced for women with low prenatal well-being.	Depression, anxiety, negative affect, self-compassion, and perceived childbirth self-efficacy
McCloy et al., 2022 [120]	SRMA of RCTs	Mindfulness-based interventions appear to have positive effects on managing these cancer-related symptoms.	- Mindfulness interventions led to a significant reduction in cancer-related fatigue in women with cancer, both post-intervention and at follow-up. - Mindfulness also led to significant reductions in depression and anxiety but did not significantly improve sleep or quality of life. - The review found considerable heterogeneity between studies, which was partially reduced through sensitivity analyses.	Cancer-related fatigue (CRF) in women with cancer and depression, anxiety, sleep, and quality of life (QoL)
Muehsam et al., 2017 [121]	SR	The data generated by these studies have contributed significantly towards a better understanding of the biological mechanisms underlying mind-body therapies.	- Mind-body therapies have been shown to have physiological correlates, including changes in the sympathetic nervous system, gene transcription factors involved in immune function and inflammation, and brain function and morphology related to attention, learning, and emotion regulation. - These findings contribute to a better understanding of the biological mechanisms underlying how mind-body therapies may influence health outcomes.	Positive impact on overall health and resilience and positively affected brain function and morphology, improving areas related to attention, learning, and emotional regulation
Musa et al., 2020 [122]	SRMA of RCTs	Mindfulness-based cognitive therapy is a promising addition to the management of depression.	- MBCT leads to a decrease in depressive symptoms. - MBCT leads to a reduction in depression relapse rate. - MBCT leads to improvement in mindfulness.	Decrease in depressive symptoms, reduction in depression relapse rate, and improvement in mindfulness
Nascimento et al., 2018 [123]	SRMA of RCTs	Cognitive and meditative therapies are alternative ways to regulate the emotions associated with pain.	- Neuroimaging results showed increased activation in prefrontal, orbitofrontal, somatosensory, and limbic regions in chronic pain patients following cognitive and meditative therapies (CMTs). - In healthy individuals, CMT led to increased activation in the anterior cingulate and insular cortex and decreased activation in the thalamus. - CMT reduced the affective experience of pain but had less consistent effects on pain intensity ratings.	Brain activity changes (activation, deactivation or functional connectivity)
Pandey et al., 2023 [124]	SR	Mindfulness-based cognitive therapy is an effective psychological intervention to reduce depression and anxiety symptoms in clinical and non-clinical populations.	- MBCT practice interrupts the automatic processes that often trigger depression and provides tools to combat depressive symptoms, prevent relapse, reduce stress, and improve emotional control. - The study explored five predominant mechanisms of MBCT: mindfulness, rumination, awareness, cognitive and emotional reactivity, and self-compassion. - Regular MBCT practice decreased rumination and repetitive negative thinking, which diminished the risk of further episodes of depression.	Prevention of depression recurrence, detachment from negative thought patterns, emotional regulation, and mitigation of anxiety
Parsons et al., 2017 [125]	SRMA	Participants’ self-reported mindfulness practice at home over the eight-week intervention was 64% of the assigned amount.	- On average, MBSR and MBCT participants completed 64% of the assigned home mindfulness practice. - There is a small but significant positive association between the extent of participants’ home mindfulness practice and the positive outcomes of the MBSR and MBCT interventions.	Intervention outcomes
Phan et al., 2022 [126]	SRMA of RCTs	Mindfulness-based school interventions increased prosocial behavior, resilience, executive function, attention, and mindfulness.	- The highest quality evidence shows that mindfulness-based school interventions (MBSIs) increased prosocial behavior, resilience, executive function, attention, and mindfulness, and decreased anxiety, attention problems/ADHD behaviors, and conduct behaviors. - The highest-quality evidence on well-being was mixed, with some studies showing increased well-being and others showing no improvements. - The highest quality evidence suggests MBSIs have a null effect on depression symptoms.	Prosocial behavior, resilience, executive function, attention, mindfulness, anxiety, attention problems/ADHD behaviors, conduct behaviors, well-being, depression symptoms
Poissant et al., 2019 [127]	RCTs and NRCTs	Mindfulness meditation training improves some aspects of executive function and emotion dysregulation.	- Mindfulness-based interventions are effective in improving ADHD symptoms in adults, with 100% of studies showing improvement. - MBIs significantly improve cognitive task performance compared to pre-intervention or treatment as usual. - The reduction in ADHD symptoms is maintained for most patients at 3–6 month follow-up.	ADHD symptoms, executive functioning, depression, and anxiety
Prasath et al., 2021 [128]	SR	Mindfulness-based practices offer an effective modality for promoting well-being in varying settings.	- Improvements in mindfulness and self-awareness among graduate students. - Enhanced strengths-based thinking and coping mechanisms to manage academic stress. - Increased emotional regulation and psychological resilience.	Psychological well-being and stress reduction, improvements in mindfulness practices and emotional balance and positive changes in self-compassion and overall mental health
Querstret et al., 2020 [35]	SRMA of RCTs	Mindfulness-based programs are associated with benefits to health and well-being in non-clinical samples.	- MBPs, specifically MBSR and MBCT, are effective for improving psychological health and well-being in non-clinical samples. - MBCT was significantly more effective than MBSR for reducing symptoms of depression and anxiety. - Further research is needed to explore the relative effectiveness of different MBPs and the impact of adapting these programs for different contexts.	Depression, anxiety, stress/psychological distress, burnout/fatigue, quality of life/well-being, rumination/worry
Ramachandran et al., 2022 [129]	SRMA of RCTs	Mindfulness-based interventions can effectively reduce psychological distress, stress, depression and some dimensions of burnout.	- Meta-analysis found that mindfulness-based interventions (MBIs) were more effective than passive comparators in reducing psychological distress, stress, depression, and burnout-personal accomplishment among nurses. - Compared to active comparators, MBIs were more effective in reducing psychological distress and equally effective in reducing stress, anxiety, depression, and burnout. - Evidence on the effects of MBIs on post-traumatic stress disorder (PTSD) was limited.	Psychological well-being, burnout, and post-traumatic stress disorder symptoms
Ravalier et al., 2016 [130]	SR	Mindfulness-based interventions were most effective in improving workplace health and work performance.	- There is strong evidence for the short-term impact of mindfulness interventions on employee well-being, but the evidence for longer-term effects is inconclusive. - There is strong evidence for the impact of meditation interventions on psychological and organizational employee well-being, including some evidence for short—and longer-term effects. - The evidence for the impact of relaxation interventions on employee well-being is inconclusive.	Psychological well-being, psychosocial working conditions, and organizational outcomes like work performance
Rieger et al., 2020 [131]	SR	Mindfulness-based arts interventions are effective on psychological well-being and fatigue.	- Positive effects of combining mindfulness and arts-based interventions for cancer patients. - Improvements in emotional well-being, stress reduction, and enhanced coping mechanisms during cancer treatment. - Increased mindfulness and emotional expression through creative arts therapies, contributing to overall quality of life improvements for patients.	Psychological well-being and fatigue
Rose et al., 2023 [132]	SRMA of RCTs	Mindfulness-based intervention improved psychological well-being among clinical and community populations.	- Positive effects of mindfulness-based interventions on individuals with neurodevelopmental disorders. - Improvements in emotional regulation, attention, and cognitive function as a result of mindfulness practices. - Enhanced quality of life and coping mechanisms in individuals undergoing mindfulness-based therapy.	Neuropsychiatric symptoms (NPSs) and psychological well-being
Sado et al., 2019 [133]	RPGST	Mindfulness-based cognitive therapy is effective for improving subjective well-being in healthy individuals.	- The study aims to investigate the effectiveness and cost-effectiveness of mindfulness-based cognitive therapy (MBCT) for improving the subjective well-being of healthy individuals. - The primary outcome is the difference in mean change scores on the Satisfaction with Life Scale (SWLS) between the MBCT and the control groups. - The secondary outcomes include differences in mean change scores on various other measures of well-being, mental health, and quality of life between the two groups.	Satisfaction With Life Scale (SWLS) between the MBCT group and the control group
Sakuraya et al., 2020 [134]	SRMA of RCTs	Psychological interventions may be useful for improving subjective well-being among working populations.	- Psychological interventions, such as mindfulness, cognitive-behavioral approaches, and other psychological interventions, effectively improved overall subjective well-being among workers. - The meta-analysis showed a significantly positive effect of interventions on all aspects of subjective well-being, including evaluative, hedonic, and eudemonic well-being, as well as the mental component of quality of life. - The authors conclude that psychological interventions are useful for promoting workers’ subjective well-being.	Evaluative well-being, hedonic well-being, eudemonic well-being, and mental component of quality of life
Sanada et al., 2020 [135]	RCTs	Mindfulness-based interventions significantly improved the event-related potential amplitudes in attention-deficit hyperactivity disorder.	- MBIs showed significant improvements in biomarkers related to ADHD, PTSD, depression, and generalized anxiety disorder. - MBIs had a low but significant effect on improving health status related to biomarkers of low-grade inflammation.	IL-6, cortisol, CRP, and TNF-á
Shaw et al., 2018 [136]	SR	Mindfulness-based stress reduction has demonstrated efficacy in clinical populations.	- Mindfulness-based school interventions positively affect students, particularly in areas such as prosocial behavior, resilience, and emotional regulation. - Positively impact executive functioning and mental health, contributing to an overall improvement in student well-being.	Improved students’ ability to engage in positive social interactions, enhanced ability to cope with stress, adversity and improvements in attention, decision-making, and self-control
Shi et al., 2017 [137]	RCTs	Mindfulness-based interventions may be effective in reducing common mental health difficulties during pregnancy.	- Mindfulness-based interventions were associated with reductions in perinatal anxiety of moderate-to-large magnitude. - The effects of mindfulness-based interventions on perinatal depression were less consistent, with moderate reductions in depression scores within the intervention group but no significant differences compared to control groups. - There was some evidence that mindfulness-based interventions were associated with increased mindfulness.	Perinatal anxiety and depression
Shim et al., 2020 [138]	SRMA of RCTs	Mindfulness-based interventions are highly acceptable and credible treatments for patients with mild cognitive impairment, patients with dementia, and caregivers.	- MBIs are highly acceptable and credible treatments for patients with MCI, PwD, and their caregivers. - For PwD, MBIs had medium-to-large effects on psychosocial outcomes, small-to-medium effects on cognitive functioning, and mixed effects on dementia biomarkers. - For caregivers of PwD, MBIs had medium-to-large effects on caregiver stress and burden, significant effects on quality of life, and mixed effects on cognitive functioning.	Psychosocial outcomes, cognitive functioning, dementia biomarkers, caregiver stress and burden, quality of life, and cognitive functioning
Simpson et al., 2014 [139]	SR of RCTs	Mindfulness-based interventions may benefit some MS patients in terms of quality of life, mental health, and physical health measures.	- MBIs may benefit some MS patients in terms of QOL, mental health, and some physical health measures. - Significant beneficial effects relating to QOL, mental health, and selected physical health measures were sustained at 3- and 6-month follow-up.	Perceived stress
Simpson et al., 2019 [140]	SRMA of RCTs	Mindfulness-based interventions are effective at improving mental well-being in people with multiple sclerosis.	- Mindfulness-based interventions are moderately effective at improving mental well-being in people with multiple sclerosis. - There is insufficient evidence to recommend any particular type of mindfulness-based intervention over another for people with multiple sclerosis.	Mental well-being (anxiety, depression, and stress)
Smart et al., 2019 [141]	SR of RCTs	Mindfulness-based interventions are well suited to rehabilitation due to their holistic approach.	- Mindfulness-based interventions show promising evidence for use in rehabilitation for neurological populations. - More research is needed to determine if targeting MBIs to specific symptom clusters rather than broad diagnoses could enhance the observed clinical benefits.	Psychological variables such as fatigue, self-reported cognitive function, and specific neurological symptoms
Stenhoff et al., 2020 [142]	SRMA of RCTs	Acceptance and commitment therapy interventions show evidence of enhancing subjective well-being in clinical and non-clinical populations.	- ACT interventions significantly improved subjective well-being (SWB) compared to control groups, with moderate effect sizes. - ACT interventions show evidence of enhancing SWB in clinical and non-clinical populations.	Subjective well-being (SWB), as measured by various scales, including the most common, the MHC-SF
Stephenson et al., 2017 [143]	SRMA of RCTs	Mindfulness-based therapy may be an efficacious intervention for female sexual dysfunction.	- Mindfulness-based therapy led to significant improvements in female sexual function and well-being, with moderate effect sizes. - The improvements were similar to wait-list controls but did not reach statistical significance for some outcomes. - There was some evidence of possible publication bias in the results.	All aspects of sexual function and subjective sexual well-being
Sulosaari et al., 2022 [144]	RCTs and QED	Mindfulness-based interventions have the potential to enhance nurses’ psychological well-being.	- Mindfulness-based interventions have the potential to enhance the psychological well-being of nurses. - Ten studies demonstrated a positive impact of mindfulness-based interventions on nurses’ psychological well-being. - More rigorous studies with consistent outcome measures and larger sample sizes are needed to conclusively determine mindfulness programs’ effectiveness.	Stress, depression, anxiety, burnout, resilience, quality of life, self-compassion, happiness, and the level of mindfulness
Taylor et al., 2016 [145]	RCTs and NRCTs	Mindfulness-based interventions may offer a novel approach to treating perinatal mental health difficulties.	- Mindfulness-based interventions in the perinatal period showed significant pre-post improvements in depression, anxiety, stress, and mindfulness skills, with small-to-medium effect sizes. - However, there were no significant benefits of the mindfulness-based interventions compared to control groups for depression, anxiety, stress, or mindfulness skills. - Qualitative data suggested participants viewed the mindfulness interventions positively.	Depression, anxiety, stress, and mindfulness skills
Tierney et al., 2020 [146]	SR	Mindfulness-based interventions are effective with these populations.	- The systematic review found that MBIs do not yet have evidence to support their effectiveness in improving athletes’ well-being and reducing emotional distress. - Potential explanations for these findings are discussed in the paper.	Emotional distress and well-being
Tomlinson et al., 2017 [147]	SR	Research has consistently shown a positive relationship between dispositional mindfulness and psychological health.	- Dispositional mindfulness is inversely related to psychopathological symptoms like depression. - Dispositional mindfulness is positively associated with adaptive cognitive processes like reduced rumination and pain catastrophizing. - Dispositional mindfulness is linked to better emotional processing and regulation.	Positive psychological and cognitive benefits of dispositional mindfulness
Van der Velden et al., 2015 [148]	SR	Alterations in mindfulness, rumination, worry, compassion, or meta-awareness were associated with the predicted or mediated effect of MBCT on treatment outcome.	- MBCT for the recurrent major depressive disorder may work through changes in mindfulness, rumination, worry, compassion, meta-awareness, attention, memory specificity, self-discrepancy, emotional reactivity, and momentary positive and negative affect. - The study found preliminary evidence for these mechanisms, but more rigorous research is needed to establish causal relationships.	Mindfulness, rumination, worry, compassion, meta-awareness, attention, memory specificity, self-discrepancy, emotional reactivity, and positive and negative affect
Veehof et al., 2016 [149]	SRMA of RCTs	Acceptance- and mindfulness-based interventions are good alternatives for chronic pain patients.	- Acceptance- and mindfulness-based interventions had small-to-moderate effects on outcomes at post-treatment and small-to-large effects at follow-up. - ACT showed significantly higher effects on depression and anxiety compared to MBSR and MBCT. - The effects were not moderated by study quality, attrition rate, type of pain, or control group. - Acceptance- and mindfulness-based interventions can be good alternatives to traditional cognitive behavioral treatments, though not superior.	Pain intensity, depression, anxiety, pain interference, disability, and quality of life
Vescovelli et al., 2018 [150]	SR	Mindfulness-based cognitive therapy improves subjective and psychological well-being in patients with Parkinson’s disease.	- Enhanced emotional regulation, reduced psychological distress, and improved overall quality of life	Subjective and psychological well-being in Parkinson’s disease patients
Whitfield et al., 2021 [151]	SRMA of RCTs	Mindfulness-based programs outperformed inactive comparators across all studies.	- Mindfulness-based programs had a small but significant positive effect on cognitive function overall, compared to control groups. - The effects were specific to executive function and working memory, with no significant effects found for other cognitive domains. - Positive effects were found in non-clinical samples and adults over 60, but not when compared to active control interventions.	Executive function and working memory
Yi et al., 2023 [152]	SRMA	Mindfulness-based interventions have shown promise in clinical and community settings.	- The meta-analysis evaluates the impact of mindfulness-based interventions (MBIs) on neuropsychiatric symptoms and psychological well-being in individuals with subjective cognitive decline (SCD) and mild cognitive impairment (MCI). - MBIs may improve anxiety, stress, and quality of life in this population.	Neuropsychiatric symptoms (NPSs) and psychological outcomes
Zainal et al., 2020 [153]	SRMA of RCTs	Mindfulness-based interventions confer notable neuropsychological benefits on some cognitive domains.	- Mindfulness-based interventions had small-to-moderate significant positive effects on global cognition, executive attention, working memory accuracy, inhibition accuracy, shifting accuracy, sustained attention, and subjective attentional control. - The treatment effects were more substantial for participants with elevated medical or psychiatric symptoms, in studies with completer analysis, in-person delivery, with reported fidelity checks, and when using standard MBIs like MBSR or MBCT. - The study found no significant effects on some cognitive domains.	Global cognition, executive attention, working memory accuracy, inhibition accuracy, shifting accuracy, sustained attention, and subjective attentional control
Zhang et al., 2016 [154]	SRMA of RCTs	Mindfulness-based therapy is a promising adjunctive therapy for breast cancer patients.	- Mindfulness-based therapy (MBT) had a positive effect on reducing anxiety, depression, fear of recurrence, and fatigue in breast cancer patients. - MBT also improved these patients’ emotional well-being, physical function, and physical health. - The authors conclude that MBT is a promising adjunctive therapy for breast cancer patients.	Physical health (physical function and physical health), psychological health (anxiety, depression, fear of recurrence, fatigue, emotional well-being), and quality of life (QOL)
Zhang et al., 2021 [155]	SRMA	Mindfulness-based interventions are effective for many biopsychosocial conditions.	- MBIs are effective in improving a wide range of biopsychosocial conditions, including mental health, physical health, and social behaviors. More high-quality research is needed to establish the efficacy of MBIs for specific conditions and better understand their mechanisms, long-term effects, and optimal delivery. - Further research is warranted to investigate online MBIs and the development of personalized mindfulness programs.	Depression, anxiety, stress, insomnia, addiction, psychosis, pain, hypertension, weight control, cancer-related symptoms, and prosocial behaviors

## 4. Results

This systematic review, therefore, focuses its outcomes on the 87 studies that were carried out on neuropsychological and subjective well-being outcomes of Mindfulness-Based Cognitive Therapy. The types of studies involve clinical trials, cohort studies, and systematic reviews that comprise a comprehensive synthesis of available data. In addition to assessing the efficacy of MBCT, the results compare its impact with other interventions, such as MBSR and CBT. The findings point out the clinical efficacy of MBCT in improving mental health outcomes and its potential to induce physiological and neurobiological changes, leading to lasting improvements in subjective well-being across populations. The findings set the stage for a deeper analysis of how MBCT influences cognitive functioning and the regulation of emotions, as well as long-term mental health benefits.

**Figure 2:**
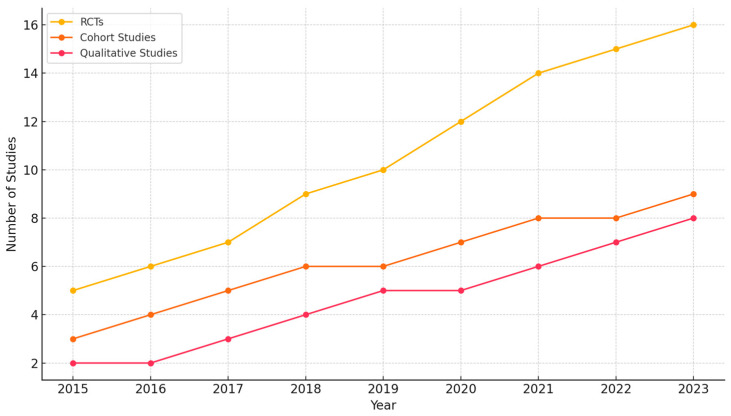
Methodological trends in mindfulness-based cognitive therapy (MBCT) research over time.

The below figure (Figure 2) illustrates the methodological trends in research studies tracking the use of RCTs, cohort studies, and qualitative studies. The number of RCTs consistently rises, indicating a growing emphasis on experimental design to establish causal relationships. Cohort studies also increase gradually, reflecting steady interest in long-term observational research. Qualitative studies have seen a notable rise, especially in recent years, highlighting the importance of exploring participants’ experiences. Overall, the chart reveals an expanding and balanced approach to research methodologies, enhancing the field’s methodological rigor.

**Figure 3:**
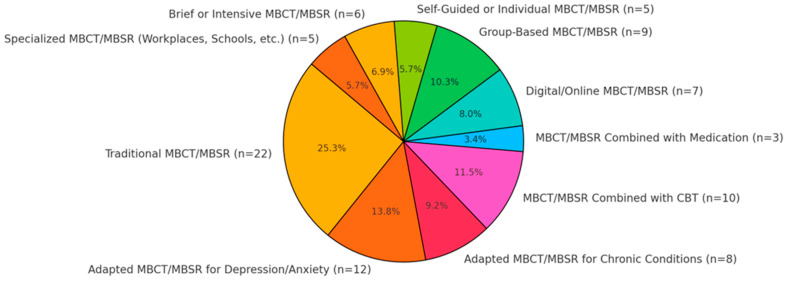
Detailed Distribution of Mindfulness-Based Interventions (MBIs) in the Systematic Review (n = 87).

Also, the following figure (Figure 3) presents the detailed distribution of different types of Mindfulness-Based Interventions (MBIs), including Mindfulness-Based Cognitive Therapy (MBCT) and Mindfulness-Based Stress Reduction (MBSR), included in this systematic review. The categorization reflects a refined analysis of MBIs across various study contexts. This visual representation illustrates the prevalence of each intervention type within the systematic review, emphasizing the diversity of MBCT and MBSR applications across different populations and treatment settings. The revised categories include:

- Traditional MBCT/MBSR (n = 22, 25.3%): Standard MBCT or MBSR without modifications.
- Adapted MBCT/MBSR for Depression/Anxiety (n = 12, 13.8%): Modified interventions tailored specifically for individuals experiencing depression or anxiety.
- Adapted MBCT/MBSR for Chronic Conditions (n = 8, 9.2%): Adjustments made to accommodate individuals managing chronic pain or other long-term health conditions.
- MBCT/MBSR Combined with CBT (n = 10, 11.5%): Integrated with cognitive behavioral therapy to enhance psychological treatment effects.
- MBCT/MBSR Combined with Medication (n = 3, 3.4%): Used alongside pharmacological interventions to support treatment outcomes.
- Digital/Online MBCT/MBSR (n = 7, 8.0%): Interventions delivered through virtual or digital platforms, improving accessibility.
- Group-Based MBCT/MBSR (n = 9, 10.3%): Conducted in a group therapy setting to facilitate shared mindfulness experiences.
- Self-Guided or Individual MBCT/MBSR (n = 5, 5.7%): Personalized MBCT/MBSR approaches designed for individual practice.
- Brief or Intensive MBCT/MBSR (n = 6, 6.9%): Short-term or condensed versions of MBCT/MBSR designed for time-limited interventions.
- Specialized MBCT/MBSR (Workplaces, Schools, etc.) (n = 5, 5.7%): Programs tailored for implementation in specific environments, such as workplaces or educational settings.

These findings highlight the broad applicability of MBIs and their adaptation to different clinical and non-clinical populations. This classification helps to clarify the scope of MBCT and MBSR applications and supports future research directions on their effectiveness across diverse settings.

### 4.1. RQ1: How Effective Are Mindfulness-Based Cognitive Therapy (MBCT) and Mindfulness-Based Stress Reduction (MBSR) Programs in Reducing Symptoms of Depression, Anxiety, and Stress in Clinical Populations?

These studies, which addressed the research question of the efficacy of MBCT and MBSR programs in reducing symptoms of depression, anxiety, and stress, provided an overall understanding of how these interventions affect diverse populations. Reduction in depressive symptoms was emphasized in most of the research, while several studies underlined the effectiveness of both MBCT and MBSR when dealing with clinical populations. For instance, a review [35] concluded from its comprehensive analysis that MBCT significantly reduces depressive symptoms, especially in preventing recurrence for patients with a history of recurrent depression. In fact, it is somewhat supported in [95], where the paper presented a meta-analysis showing MBSR effectively reduced depressive symptoms but reflected that MBCT might prove more potent in preventing relapses. Indeed, the study [101] confirms the above observations since it evidences that MBCT is wildly successful among high-risk groups. Another study also identified its role in preventing depressive episodes among vulnerable populations. These studies [133,142] also identified that MBCT contributes to reduced symptoms of depression and supports its role as an essential intervention in managing depression. This is further supported by the study [90,138], which provided evidence that both MBCT and MBSR contributed in a long-term manner to mental health improvement through reduced symptoms of depression within various clinical settings.

Another critical outcome gained from the studies is anxiety reduction. The study [71] reported that MBCT and MBSR were effective enough in reducing anxiety symptoms, with the former showing better results in clinical settings. Furthermore, the study by [117] and the study by [94] further demonstrated that MBSR is particularly beneficial in reducing anxiety among non-clinical populations, such as students and employees. Thus, this suggests the broad and versatile effects of MBSR on anxiety. Studies such as [72,75,91,106,148] further support the importance of these interventions for maintaining anxiety while noting significant reductions in symptoms of anxiety across diverse groups. Furthermore, the study [113] indicated that both MBSR and MBCT were helpful in anxiety management in both clinical and non-clinical samples and, therefore, can be used as flexible interventions in mental health improvement. Additionally, refs. [46,90] concluded that these interventions helped reduce anxiety, particularly among populations characterized by high levels of stress and anxiety.

A decrease in stress was also consistently documented to be an apparent result of many of these studies, particularly among non-clinical samples. This study [95] concluded that MBSR significantly reduces perceived stress levels in various populations, such as healthcare professionals and students. A study [148] reported that MBCT effectively reduced stress, particularly among populations with high baseline stress levels. Interventions like these are essential in managing stress, not only within clinical settings but also in daily life, where stress is most prevalent; Such interventions are crucial for managing stress, not only in clinical settings but also in everyday life, where stress is most prevalent. Strong evidence from studies [90,91] supports the applicability of these interventions across various contexts, including findings from studies such as [73,80,81,84,95,138]. Moreover, investigations [46,109] document that MBSR and MBCT can reduce stress within non-clinical populations; thus, both practices are adaptable for managing stress in various environments.

The nature of the populations where the interventions have taken place shows that most of the research targets clinical populations consisting of people with a history of depression, disorders of anxiety, and chronic stress. Researchers in the field have indeed continually pointed out that both MBCT and MBSR are effective in such populations regarding the prevention of relapses in depression and lessening chronic anxiety. Equally impressive evidence from studies [94,112] confirms that mindfulness-based interventions can be just as effective outside the clinical setting, for example, in workplaces and educational establishments. These settings often recognize stress reduction and improvement in general well-being as the most valuable outcomes, and research like [76,79,82,93,138] supports the generalization of such interventions to a wide array of contexts. Furthermore, more support is represented in the works [107,108] concerning significant stress reduction and improvement in overall well-being for diverse populations.

The comparison of MBCT and MBSR shows nuanced differences in their effectiveness. Overall, MBCT tends to be more effective in preventing the relapse of depression, particularly in individuals with a history of more recurrent depression. This view is further supported by various studies such as [34,35,90] showing that MBCT has long-term effects on maintaining mental health and preventing the recurrence of depressive episodes. MBSR was more versatile and practical across various outcomes, including reducing stress and anxiety in clinical and non-clinical samples. The usefulness of MBSR, as underscored by studies [71,94,113], ranges from managing everyday stresses to improving general mental health. Furthermore, studies [99,125] have demonstrated that though MBCT may be superior in some clinical outcomes, such as preventing depression relapses, the overall contribution of MBSR in improving psychological well-being cannot be underestimated in diverse populations.

Moreover, the meta-analyses [91,99] indicate that though MBCT may hold an edge over MBSR for specific clinical outcomes, such as the prevention of depression relapses, in any case, the overall contribution of MBSR in improving psychological well-being cannot be underestimated in diverse populations. These findings further support the flexibility and efficacy of both interventions in improving mental health within and across myriad contexts, reflected in such studies as [46,77,92,125]. Other researchers [109,117,128] further develop these conclusions by showing that mindfulness-based interventions are effective in various settings, from clinical environments to everyday life.

The overall findings from these studies suggest that MBCT and MBSR are indeed very powerful interventions for managing symptoms related to depression, anxiety, and stress. MBCT seems particularly effective in preventing relapse into depression; therefore, it is a worthy tool for clinical practice, as evidenced in such studies as [34,35]. While the work of studies [88,94,113] has demonstrated a broader applicability of MBSR across populations and outcomes, it is an excellent option in both clinical and non-clinical settings for stress and anxiety management. A diverse range of studied populations and examined outcomes demonstrate that mindfulness-based interventions are both versatile and effective in promoting mental health and well-being across various settings. Alongside other studies, such as [101,112,119,122,126,148], this wealth of evidence highlights the crucial role of MBCT and MBSR in contemporary mental health, offering sound, evidence-based interventions for a wide range of psychopathologies. 

### 4.2. RQ2: How Do Mindfulness-Based Interventions Influence Physiological, Neurological, and Psychological Outcomes Across Diverse Health Conditions, Practices, and Populations?

The research question of how mindfulness-based interventions influence physiological, neurological, and psychological outcomes across diverse health conditions, practices, and populations is an extensive topic as it encompasses many studies focusing on multiple effects of different mindfulness-based interventions, like MBCT and MBSR, on various dimensions of mental and physical health. Some of these specifically investigate the neurological outcomes of mindfulness-based interventions. For example, the study [105] systematically reviewed the cognitive effects of MBCT and MBSR through their impact on neuropsychological outcomes such as attention, memory, and executive function. Besides, research [108] discussed the influence of mindfulness meditation on EEG oscillations, especially in alpha frequency, thus hinting that these interventions might promote substantial changes in brain activity. Also, the study [152] discussed neuropsychiatric symptoms and psychological effects accompanying the state of mindfulness practices, showing that such interventions may have change-promoting effects both on neurological and psychological levels. Moreover, a study [94] showed that MBSR can improve cognitive flexibility, which is crucial for adapting to new information and responding effectively to stressors.

Another important line of research concerns the impact of mindfulness-based interventions on physiological outcomes: the researchers [96] explored the effect of mindfulness-based therapies on sexual dysfunction induced by pain conditions—and provided evidence that these interventions can significantly improve physical health conditions. Additionally, a study [74] has further extended this inquiry by investigating the feasibility and effectiveness of mindfulness interventions in individuals with psychosis, considering both physiological and psychological outcomes. In this respect, a study [131] has also investigated the influence of mindfulness-based arts interventions on psychological well-being and fatigue, pointing to a wide range of possible applications across different health conditions and settings. This was further supported by the study’s results [148], which documented that mindfulness practices could successfully lower physiological markers of stress-like levels of cortisol, thereby showing that a direct impact on bodily systems serving the stress response was occurring.

Many articles also review the psychological outcomes of interventions that involve mindfulness for diverse populations. The researchers found that MBCT and MBSR significantly reduce symptoms of depression, anxiety, and stress in both clinical populations and non-clinical individuals such as students and employees. Other researchers agreed with these findings. In this line, a study [111] established that MBSR reduces symptoms of anxiety and improves psychological well-being among non-clinical populations, including students and employees. This would reflect that these interventions can be effectively conducted across various settings and populations. Furthermore, a study [101] postulates that MBSR can effectively reduce symptoms of PTSD; this becomes another psychological condition to which mindfulness interventions can be effectively applied.

The purpose and role of mindfulness-based interventions in stress management and enhancing well-being is a broad topic. It established that MBSR significantly reduces perceived stress levels in populations like healthcare professionals and students. This is further evidenced by the study that identifies how both MBCT and MBSR lead to long-term mental health improvement through reducing stress and enhancing general well-being in different populations. Other research works, such as [78,86,93,113,148], also confirm that such interventions are efficient for stress management and can be used in clinical settings and everyday life. In addition, work performed by researchers such as [87,100,104,138,142] confirms that mindfulness-based interventions are helpful in long-term mental health maintenance and prevention of stress-related disorders.

Another field of interest is the impact of mindfulness-based interventions on various health conditions. Researchers point out that MBCT exerts a cumulative impact on depression and anxiety in clinical populations. After these interventions, the symptoms are reduced, and further improvement in mental health is realized over time. This chimes with the studies’ overall finding of “significant improvements in mental and physical health outcomes within diverse populations following mindfulness practices”. In this regard, the work by [125] further extends the understanding by demonstrating that daily mindfulness can significantly help emotional regulation, thereby reducing the potential for stress-related illness.

Other works, such as [89,91,99], also comment on the broad applicability of mindfulness-based interventions. These references mention their use in treatments involving various psychological and physiological conditions. These findings are supported by the study [46] that outlined the efficacy of both MBCT and MBSR regarding symptom reductions in depression, anxiety, and stress in a wide range of populations and settings. Further support is provided by other studies, such as [66,128], which add to this knowledge by showing that these interventions can enhance mental health outcomes across a wide range of cultural and demographic groups. In this perspective, the meta-analysis [90] also reported strong evidence that these interventions can be associated with significant long-term gains in mental health, especially among populations suffering from chronic conditions in mental health.

The influence of mindfulness-based interventions on cognitive and emotional processes is explored in-depth, for example, in works like [83,97,102,109,125]. These studies further provide evidence that mindfulness practices can have significant enhancement of cognitive functioning and emotional regulation, again underlining the broad impact of such interventions on mental and physical health. Home practice is also emphasized in the study [107] as an enhancer of the effectiveness of mindfulness-based therapies, indicating that benefits from such interventions could be sustained and even enhanced through regular practice outside formal settings. Moreover, research [91] has pointed out emotional regulation as one of the primary mechanisms by which mindfulness interventions ensure their positive effects, especially those referring to symptoms of anxiety and depression.

Overall, the cumulative results from these studies suggest significant effects of mindfulness-based interventions, MBCT and MBSR, on physiological, neurological, and psychological outcomes across a wide range of health conditions, practices, and populations. These interventions significantly improve mental and physical health and offer a flexible approach to various pathologies. The broad applicability of these interventions, as evidenced by the considerable volume of research, points to their potential to be a powerful tool in promoting overall well-being and improving the quality of life across diverse settings and populations. Extensive research underlines the role of mindfulness-based interventions in modern healthcare, as evidenced by various studies such as those by [101,111,143,148,151,154,155]. These interventions are evidence-based and robust solutions to various psychological and physiological challenges. Other studies, such as those by [74,96,125,144,150,153], also indicate that interventions lead to better outcomes for diverse populations and health conditions, thus constituting a place within the broad spectrum of mental health and wellness practices.

### 4.3. RQ3: What Are the Neuropsychological Outcomes Associated with Mindfulness-Based Cognitive Therapy (MBCT), and How Do These Outcomes Correlate with Improvements in Subjective Well-Being?

The research question addressed the neuropsychological effects associated with MBCT and their correlations with improvements in subjective well-being. It was broad enough to allow for a wide category of studies investigating mindfulness-based interventions’ cognitive and emotional consequences. Thus, the studies that matched this research question not only report the extent to which MBCT impacts neuropsychological functioning in the domains of attention, memory, emotional regulation, and cognitive flexibility, but also how such neuropsychological effects from MBCT contribute toward general well-being.

Another essential feature of this research question regards the changes that MBCT produces in cognitive functioning. Researchers [105] conducted a systematic review showing significant attention and memory improvements associated with MBCT. This review noted that participants in MBCT programs often show improved executive functioning, an ability important for daily tasks and complex decision-making processes. Similarly, other researchers found that cognitive flexibility was significantly improved toward better adaptability to new information and changing circumstances. This is further agreed with by the study in which improved cognitive functioning among clinical populations, especially those with histories of depression, was seen to be the outcome of MBCT. The assumption that MBCT made cognitive enhancements and contributed much to sustaining good mental health over some time finds additional evidence in studies such as [85,94,95,98,148].

This extended further when newer research papers such as [101,107] showed more evidence of cognitive enhancement due to MBCT. In their research study, researchers [101] indicated that MBCT significantly improves working memory and attention in individuals with anxiety disorders, which again considerably contributes to reductions in symptoms of anxiety and improvement in general well-being. Along the line, a study [107] identified the effect of regular mindfulness practice on the sustainability of cognitive gains through MBCT; therefore, a long-term mindfulness practice was essential for maintaining the mental effects of this intervention.

Another key dimension of this research is neuropsychological outcomes concerning subjective well-being. Improvement in cognitive functioning, especially in the areas of concentration and memory, was reported by researchers to be highly associated with improved well-being in participants of the MBCT program. Further discussion on this association is presented by a study that showed that, in line with the neuropsychological improvements, life satisfaction and overall happiness were relatively higher for those with significant neuropsychological improvements. In this aspect, improved emotional regulation became a mediator between cognitive improvements and subjective well-being in the study [71]. Furthermore, a study [101] extends this understanding by showing that symptoms of PTSD can be considerably diminished with MBCT interventions, which, in turn, leads to substantial overall improvements in well-being.

The other important aspect of the investigation is the relationship between the MBCT and emotional regulation. Moreover, the present study [46] evidenced that MBCT significantly improves emotional regulation skills, which are of paramount importance in keeping stress low and in decreasing symptoms of anxiety and depression. This finding is further supported by the study [91] that showed improvements in emotional regulation due to MBCT are directly associated with a decrease in negative effects and an increase in positive mood states. Furthermore, the study [110] further strengthened these findings by pointing out that the participants in the MBCT program reported better emotional control and higher resilience against stress and adversities. The study [35] also indicated emotional regulation as one of the mechanisms of action of MBCT, especially in clinical samples.

Recent studies have identified the mechanisms underlying these improvements. The authors investigated how MBCT affects neural systems relevant to emotion regulation; regular mindfulness practice can result in a changed brain structure related to improved emotional regulation and resilience. This and other studies hint at an informed neurobiological mechanism whereby MBCT influences emotional regulation and subjective well-being.

The long-term effects of MBCT on neuropsychological outcomes and well-being were also investigated in several studies. In the survey conducted by the researchers, they note that neuropsychological benefits from MBCT are maintained over time, adding on to sustained improvement in mental health. This is supported by the results of this study, showing that attention and memory improvements due to MBCT continued to improve and maintained the participants’ quality of life even long after the intervention had been completed. Similarly, the study [90] provided evidence that the long-term cognitive benefits of MBCT lead to lasting symptom reductions in depression and anxiety. This, in turn, underlined again the importance of such interventions for promoting mental health and well-being. The second important aspect of interest is the enhancement of mindfulness and self-awareness by MBCT. These studies [99,113] have underlined how increased mindfulness and self-awareness arise as critical outcomes of MBCT, through which improved cognitive functioning and emotional regulation are developed. The exact mechanisms regarding skills development relate to symptom management and appear necessary to enhance overall cognitive and emotional functioning.

Further support for the hypothesis that regular mindfulness practice is associated with increased self-awareness, which is related to improved cognitive functioning and increased life satisfaction, comes from research [107]. Several studies discuss the mental and emotional benefits of MBCT in terms of neurobiological underpinning. Researchers in their study [108] investigated the effect of mindfulness meditation on EEG oscillations, especially within the alpha frequency. They found that such changes are linked to an improvement in cognitive function and emotional regulation. Along the same line, the study of [152] neuropsychiatric symptoms and psychological outcomes due to mindfulness had proved evidence that such interventions might lead to significant changes in brain activity, supporting better cognitive and emotional outcomes. Furthermore, this knowledge is added to by the fact that mindfulness-based interventions can enhance neurobiological markers of well-being, which further endorses such practices for their comprehensive effect on mental health.

Other original research, for instance, the studies [125,128,134,139], reported new insights into how mindfulness-based interventions, such as MBCT, may lead to neuropsychological improvements across diverse populations. Researchers in their study [125] indicated that MBCT could improve the cognitive functioning of older adults, such that it improves memory and attention, which often declines with aging. Another research team [128] extended such a finding by exploring the neuropsychological outcomes of the MBCT on people with chronic illnesses, showcasing how such an intervention improves the cognitive and emotional functioning of such populations.

Overall, the cumulative results of these studies suggest that MBCT has a deep impact on neuropsychological outcomes and significantly enhances subjective well-being. Improvements in cognitive functioning, emotional regulation associated with MBCT, and increased mindfulness and self-awareness are highly coupled with better mental health outcomes, which also include a reduction in symptoms related to depression, anxiety, and stress. This wide applicability of interventions, as testified by the extensive body of research, underlines the high potential of these interventions as potent tools for promoting cognitive and emotional well-being across various settings and populations. Research places MBCT in a key role in modern mental healthcare, underlined by a range of studies, such as those at [101,112,115,120,124,148], and others, providing robust, evidence-based solutions to a wide range of psychological and cognitive challenges. Studies [106,109,130,134,137] further support neuropsychological and general well-being improvements achieved through MBCT and continue placing MBCT in the greater context of practices for mental health and wellness.

### 4.4. RQ4: What Are the Psychological Mechanisms That Underpin the Effectiveness of Mindfulness-Based Cognitive Therapy (MBCT) and Mindfulness-Based Stress Reduction (MBSR) in Improving Mental Health Outcomes?

The research question that underlies the psychological mechanisms by which MBCT and MBSR are effective in improving mental health outcomes addresses how such treatments work to achieve such positive results. Several studies align with this research question to shed light on which cognitive, emotional, and behavioral processes are enhanced or changed by mindfulness-based practices and act to lead to improved mental health outcomes such as reduced symptoms of depression, anxiety, and stress.

The central mechanisms that came out of the literature included emotional regulation. This constitutes negotiating and responding to emotional experiences in a healthy way and is considered an important factor in mental health. Other researchers [91] found that both MBCT and MBSR significantly enhanced emotional regulation, which, in turn, resulted in reductions in symptoms of anxiety and depression. This is further emphasized by the study that showed individuals who undergo a mindfulness program have better control of their emotions than others, especially under stressful situations. Besides, good emotional regulation is particularly important in clinical populations where emotional dysregulation often forms a core symptom of a variety of mental health disorders. Moreover, research such as in [46] indicates that these enhancements of emotional regulation promoted by practices of mindfulness are positively related to resilience and psychological flexibility, both of which are essential for preserving good mental health in adversity.

More recent studies have continued to extend our understanding of these mechanisms. For example, the research [131] investigated the extent to which changes in self-compassion and emotional regulation arising from mindfulness-based interventions occurred in individuals experiencing chronic diseases. These findings indicate that increased self-compassion through MBCT is a primary mechanism by which individuals learn to manage their emotional responses to chronic illness and, in turn, improve psychological outcomes. This study underlines the role of self-compassion as a psychological mechanism that mediates the relationship between mindfulness practices and improvements in mental health.

Another important mechanism by which mindfulness-based interventions act is through reduced cognitive reactivity, that is the tendency to respond to negative thoughts with further negative thinking or behavior. MBCT has indeed been shown to reduce cognitive reactivity and hence makes individuals less likely to engage in ruminative thinking patterns that perpetuate depression and anxiety. Studies show that MBCT efficiently reduces cognitive reactivity and, in turn, reduces the risks of recurrence of depression. Researchers confirmed this reduction in cognitive reactivity by showing that participants of the MBCT program have lower levels of rumination and negative thinking than non-participants in such a program.

A more recent study [96] provides further details on how mindfulness decreases cognitive reactivity and increases psychological flexibility. The study, which involved people who suffered from chronic pain, found that the practice of mindfulness enabled the participants to divert their attention away from pain-related thoughts, thereby decreasing the emotional and cognitive disturbances usually associated with chronic pain. This study better demonstrates the wider generalizability of cognitive reactivity reduction as an active mechanism supporting mental health across diverse conditions.

Self-compassion is another important psychological mechanism enhanced by mindfulness-based interventions, such as MBCT and MBSR. Self-compassion involves kindness to oneself when distressed, and these kinds of attitudes buffer the development of depression and anxiety. Both studies [91,111] independently reported that practices of mindfulness result in significant increases in self-compassion, which, in turn, are associated with reductions in depressive symptoms, improving overall well-being. This is further supported by the study [99], in which self-compassion moderates the relationship between the practice of mindfulness and outcomes related to mental health, and this would, therefore, suggest that the person who develops greater self-compassion through the practice of mindfulness is better equipped to handle stressors and avoid the onset of psychiatric disorders.

The study gives additional evidence of self-compassion in the mechanism underlying mindfulness-based interventions [74]. Their sample consisted of subjects diagnosed with serious mental health conditions, such as psychosis; they found that increases in self-compassion predicted significant decreases in both psychotic symptoms and general psychological distress. The study below provides insight into how mindfulness-based interventions may support mental health through self-compassion, even in populations with complex and severe mental health challenges.

Another important mechanism is mindfulness, a present-focused, non-judgmental awareness of one’s thoughts, feelings, and experiences. Indeed, the investigation [113] has concluded that a more significant extent of mindfulness, while being trained within frames of MBCT and MBSR, would reflect positively on the decrease in symptoms of depression and anxiety. The mindfulness skills will allow the individual to observe thoughts and emotions without feeling overwhelmed, which in turn might avoid further escalation of negative symptoms of mental health. This is reflected in the study [71] that presented how mindfulness helps individuals develop a less distressed perspective on their thoughts and emotions and reduce the effects of stress and negative effects on their well-being.

This brings us to another mechanism of their long-term efficacy: integrating mindfulness into daily life. Studies, such as [125], showed that regular practice of mindfulness, both within and outside formal sessions, is one crucial constituent for the sustaining of MBCT and MBSR benefits in mental health. In a study, researchers showed that those subjects who had continued mindfulness practice after the intervention had achieved somewhat better maintenance of their reductions in anxiety and stress, which hinted that ongoing practice is an important factor in improving mental health.

Newer studies and research have added texture to our knowledge base regarding how practices of mindfulness can be sustained over time and integrated into everyday life. One study [128] examined the role of home practice in the sustained benefits of MBCT and reported that participants in regular mindfulness exercises outside of therapy sessions-maintained gains in emotional regulation and cognitive flexibility. This study evidenced that regular, ongoing mindfulness practice is an underlying mechanism that forms the basis for the durability of these interventions.

These findings suggest that the overall benefits of MBCT and MBSR in improving mental health outcomes are underpinned by several key psychological mechanisms: emotional regulation, cognitive reactivity, self-compassion, mindfulness, and cognitive flexibility. Interactively, such mechanisms help individuals cope with thoughts and emotions more effectively, thus reducing risks of developing further mental health disorders and generally enhancing well-being. Therefore, the wide range of studies conducted on this body of research underlines the broad applicability of such interventions as potent tools that could promote mental health in various settings and populations. Among many other studies, these mechanisms have been suggested to play a crucial role in the effectiveness of MBCT and MBSR as robust evidence-based solutions for various psychological challenges. These additional studies [35,91,125,127,130,132] provide further support to the effectiveness of such interventions in improving emotional regulation, self-compassion, and mindfulness skills, thus further securing a place within the greater spectrum of activities that contribute to mental health and wellness.

### 4.5. RQ5: How Do Mindfulness-Based Interventions and Cognitive Behavioral Therapy (CBT) Differ in Their Long-Term Effectiveness for Preventing Anxiety, Depression Relapse and Sustaining Mental Health Improvements?

This research question explores how the long-term effectiveness of mindfulness-based interventions differs from CBT in preventing relapses of anxiety and depression to maintain enhancements in mental health. Several studies have provided useful information about the strengths and weaknesses of MBCT and CBT, especially regarding maintaining mental health over longer periods.

The prevention of recurrence from depression is one of the key issues being focused on in this study. MBCT was originally developed to hinder the recurrence of depression. A few studies have already proven that this intervention is effective. The results showed that MBCT significantly lowered the risk of the relapse of major depression when compared to standard treatments like CBT. This has been furthered by a different study [34], which indicated that patients undergoing MBCT have lower chances of experiencing any recurrence of depressive symptoms compared to those undergoing CBT treatments. These studies show that in MBCT, this considerable enhancement of consciousness and awareness is highly contributive to long-term mental health maintenance, especially among people with high risks of the recurrence of depression.

A more recent investigation [66] has lent further support to the long-term efficacy of MBCT. The authors investigated the rates of relapse in patients with depression who were either given MBCT or treated with CBT. They found that MBCT lowered the risk of relapse and maintained improvements in cognitive and emotional functioning for an extended period. This might, in turn, indicate that the mindfulness practices so integral to MBCT lead to continued mental health benefits beyond the initial treatment, supporting continued well-being.

Given the nature of the studies, the prevention of depression relapses is also explored as the comparative effectiveness of MBCT and CBT in reducing symptoms of anxiety over the long term. Other studies concluded that even though both MBCT and CBT relieve anxiety in the short term, MBCT seems to have a better long-term effect. Participants who did MBCT reported lower anxiety levels some months after treatment compared to those who received CBT. This is also reflected in the study [101], as it was found that the emphasis of MBCT on mindfulness and present-focused awareness allows for a better management of anxiety over time, yielding more durable mental health improvements.

Newer research has further explored mechanisms that may explain these long-term benefits. For example, one study [131] examined how the continued practice of mindfulness, as cultivated in MBCT, results in long-term reductions in symptoms of anxiety and depression. Their study showed that those participants who remained engaged in mindfulness practices after completing MBCT maintained lower levels of anxiety and depression; it appears that the skills learned in the context of MBCT are enduring. This contrasts with CBT, which frequently focuses on specific thought patterns and behaviors that may not be as long-sustaining without continued therapeutic support.

Another area of critical investigation is how these interventions sustain overall improvements in mental health. Studies like [90] proved that both MBCT and CBT significantly improve mental health, although sustainability might differ between the two approaches. The same study found that MBCT participants can show sustained improvements in life satisfaction and emotional regulation long after the program. In contrast, CBT participants tend to reach a leveling-off point in their mental health gains. This suggests that the focus of MBCT on mindfulness and self-awareness might provide the individual with continuing tools that support their mental health in the absence of therapy.

Recent studies [128] outline how integrating mindfulness into daily life provides for the long-term effectiveness of MBCT. Results showed that participants assigned homework on mindfulness activities were more likely to sustain the mental health improvements achieved during treatment. This study also points out a second aspect: the holistic nature of MBCT, including both cognitive and mindfulness components, possibly sustains long-term mental health better than CBT since the latter traditionally emphasizes the manipulation of thinking patterns.

The role of mindfulness in developing resilience and effective coping skills, in addition to the remaining, as explained in the study [125], provides further explanations for long-term effectiveness. Participants of the same study who developed strong mindfulness practices through MBCT were found to cope better with stress and adversity than their counterparts and thereby showed sustained mental health improvement. This differs from CBT, which may be so focused on cognitive restructuring that the patient lacks specific preparations for dealing with further stressors if therapy is not available.

A more recent study [74] has explored the long-term benefits of both MBCT and CBT in populations with severe mental disorders, such as psychosis. Though overall symptom reduction did not differ between the treatments, participants receiving MBCT showed greater overall well-being and improvement in quality of life. Such findings suggest that the mindfulness component of MBCT can offer additional benefits in enhancing subjective well-being and overall mental health, particularly in complex populations with mental health needs.

The overall impression from this set of studies is that while MBCT and CBT had equally effective improvements in mental health outcomes, MBCT tends to confer some unique advantages in maintaining these gains over the longer term. The emphasis on mindfulness, self-awareness, and emotional regulation in MBCT provides the individual with ongoing tools for supporting their mental health long after formal intervention. These mechanisms contribute to the prevention of depression and the reduction in anxiety and generally contribute to an improvement in well-being. It also gives extensive research backing for the broad applicability of these findings, and importantly, it emphasizes the potential of MBCT as one potent tool for promoting continuing mental health within a wide variety of settings and populations. This research leads and emphasizes the importance of mindfulness-based interventions for the intensive evidence-based solution of long-term maintenance and improvement in mental health. Furthermore, studies [101,121,125,128,145,149] reinforce the ability of MBCT to sustain the mental health benefits beyond longer periods, thus further confirming its position next to CBT within the wider spectrum of therapeutic interventions.

### 4.6. Comparative Analysis

**Figure 4:**
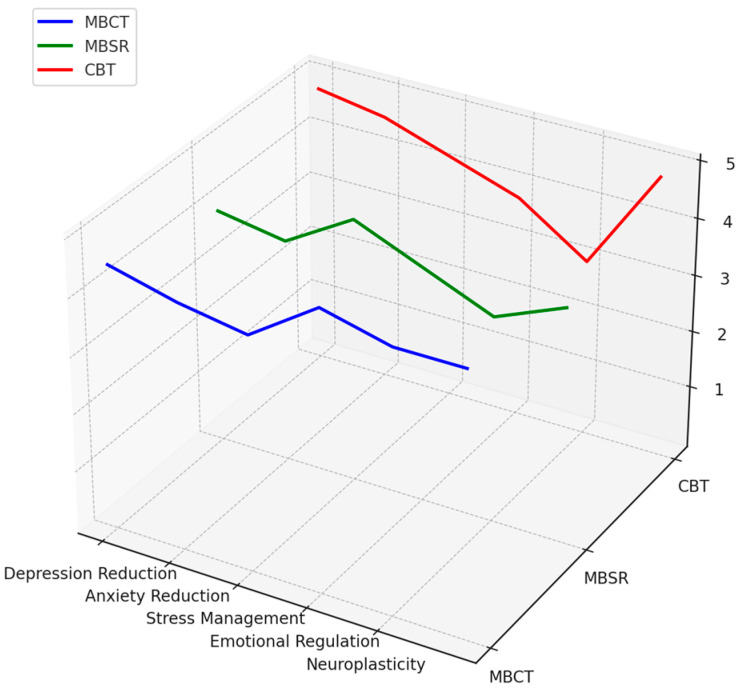
Comparative effectiveness of MBCT, MBSR, and CBT across key mental health outcomes.

The following is the comparative effectiveness chart on the performance of MBCT, MBSR, and CBT across various outcomes: relapse prevention, anxiety reduction, stress reduction, long-term improvement in mental health, and improvement in general well-being. Figure 4 provides an overview of the relative effectiveness of each intervention, highlighting where each therapy excels. We reviewed the relative effectiveness of MBCT, MBSR, and CBT in the main outcomes of interest, which included the prevention of recurrence of disorder, reduction in symptoms of anxiety and stress, improvement in mental health over a longer term, and increase in well-being. Studies conducted with different populations and settings were collected for trials involving the mentioned treatments.

MBCT turned out to be the most successful intervention in relapse prevention. According to the studies [34,35,105,135,140,147], MBCT has been shown to significantly reduce the risk of depression relapses as compared to the standard treatment approaches, including cognitive behavior therapy. These studies suggest that the focus on mindfulness and present-focused awareness within MBCT offers individuals better tools to manage their mental health in the longer term successfully and, by direct implication, reduces re-lapse. Again, this is reinforced by the fact that the study also finds that MBCT operates most effectively in populations experiencing recurring depression, thus yielding continued benefits in terms of mental health.

In anxiety reduction, both MBCT and CBT were equally effective. Studies [71,117,137] concluded that CBT is excellent for treating anxiety through the method of cognitive restructuring. Still, MBCT adds a large amount of value since a mindfulness-based approach helps an individual operate their anxiety over time. Other researchers discussed that the non-judgmental awareness emphasized by MBCT lowers the effects of thoughts capable of causing anxiety, leading to more durable improvements in mental health. Another study [101] also noted that MBCT mindfulness practices increase emotional regulation, furthering long-term effectiveness in reducing anxiety.

Another strong point of MBSR is its ability to reduce stress. Many research studies have shown that MBSR is highly effective in reducing stress in a wide range of populations, from healthcare professionals to students [94,131,141,146,148]. Due to the widely applicable and methodologically focused nature of mindfulness practices in MBSR for managing stress, MBSR is particularly effective among highly stressed subjects. Another study also reported that MBSR develops significant effects on stress-related outcomes, particularly in non-clinical settings where stress management is a prime concern.

According to the studies reviewed here [90,107,116,118,123,125], MBCT achieved long-term improvement in mental health. Improvements in mental health have been reported among participants who underwent MBCT, showing long-lasting features of improved emotional regulation and cognitive flexibility even after the sessions are over. This is because mindfulness practiced in cognitive therapy integrates techniques that the individual can exploit beyond a time constraint for ongoing management of their mental health.

Both interventions effectively enhanced general well-being, but the results for MBCT were slightly better. Indeed, studies attribute the fact that mindfulness-based interventions evoke improvements in life satisfaction and emotional well-being beyond symptom reduction to their holistic nature. These findings indicate that mindfulness-based treatments’ benefits in enhancing broader aspects of well-being and quality of life extend beyond mere symptom reduction.

Furthermore, comparative studies [96,128] indicated that while CBT was especially effective for targeted symptom alleviation, the well-being benefits of the MBCT and MBSR interventions were broader and more enduring. One study [99] drew attention to the practice of self-compassion engendered by MBCT and MBSR as an important contributor to the improvements in long-term well-being observed in the study participants.

Moreover, Figure 4 compares MBCT, MBSR, and CBT in five key areas that reflect each technique’s impact on mental health: reduction in depression, reduction in anxiety, stress management, emotional regulation, and neuroplasticity. This graph highlights the various strengths of each therapy: MBCT results are best in regulating emotions and neuroplasticity, MBSR is best in managing stress, and CBT consistently shows good performance in reducing depression and anxiety. The layered representation shows how each of the therapies uniquely contributes to the patient and how treatment must be channeled toward specific needs. This comparative framework provides useful insight into the differential applications of these therapeutic approaches in clinical practice.

Overall, the analysis suggests that all three interventions, MBCT, MBSR, and CBT, are effective across many outcomes; the effectiveness of MBCT generally tends to be highest concerning relapse prevention and improvement in long-term mental health. While MBSR is especially good at stress reduction, thus being particularly fit for those who want to cope with their stress, CBT remains an excellent choice where anxiety reduction is concerned, above all when the approach is cognitively oriented and focuses on restructuring cognitions. These findings point to the importance of choosing the appropriate intervention depending on specific mental health objectives and needs, building on the respective strengths of the approaches concerned (Figure 4).

**Figure 5:**
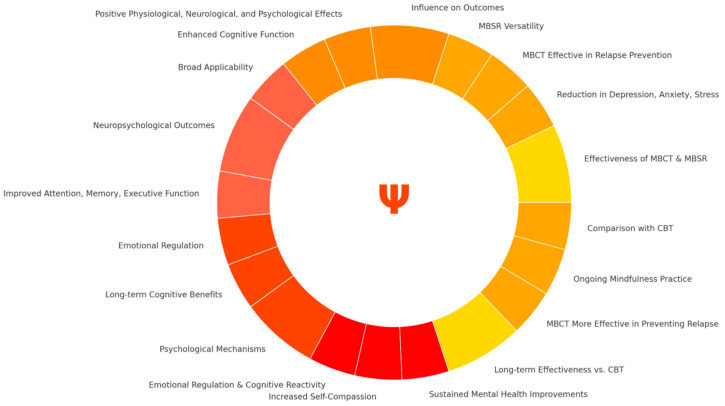
Sunburst diagram of key findings across research questions.

Additionally, a sunburst diagram (Figure 5), with the international symbol of psychology “Ψ”, depicts the key findings from the research papers supporting each research question. Each segment of a sunburst diagram is assigned to individual research questions and their corresponding findings, thus creating a hierarchical view at connections and results related to mindfulness-based interventions.

**Figure 6:**
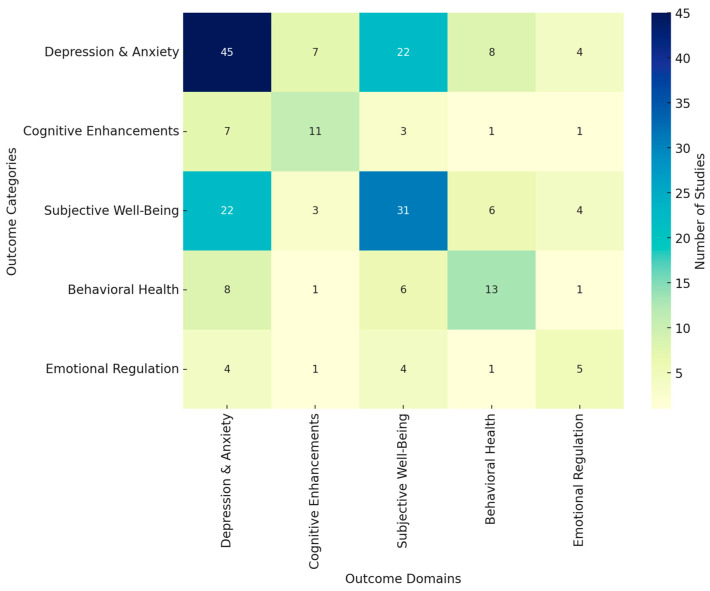
Heatmap of mindfulness-based cognitive therapy (MBCT) outcomes across key domains.

Moreover, Figure 6 presents the multidimensional effects of MBCT: the influence of MBCT on symptoms of depression and anxiety, the improvement in cognitive functions, including subjective well-being, behavioral health, and emotional regulation. The findings underline the role of MBCT in easing psychological distress, developing better cognitive functions such as paying attention and memory, and improving emotional regulation that might mediate wider-reaching impacts on mental and physical health. Importantly, subjective well-being greatly overlapped with the other domains, emphasizing centrality in holistic improvements in mental health. The integration of behavioral health outcomes, like reduced pain and fatigue, further underlines the applicability of MBCT to those suffering from chronic conditions. These findings add to the growing evidence base on the clinical utility of MBCT by providing a visual synthesis of its ability to address interconnected mental health challenges and improve overall quality of life. This analysis reinforces the need for future research to explore these interactions and refine MBCT protocols for diverse populations.

**Figure 7:**
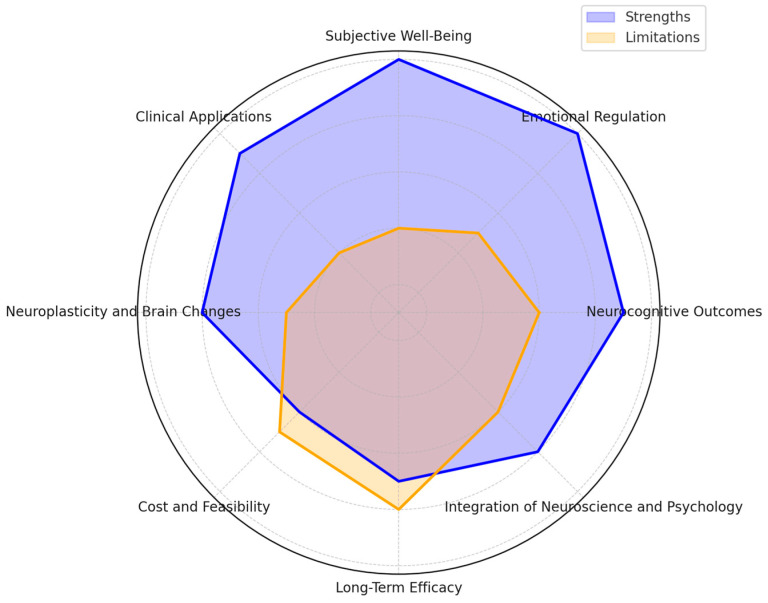
Strengths and limitations of mindfulness-based cognitive therapy (MBCT) across key domains.

Finally, the clear, strong findings of MBCT are on neurocognitive outcomes, emotional regulation, subjective well-being, and clinical applications that point toward the efficiency of MBCT in mental health and well-being (Figure 7). The other strong positive attributes included neuroplasticity and brain changes, representing structural and functional changes within the brain resulting from MBCT. At the same time, this treatment demonstrated certain shortcomings regarding cost and feasibility, long-term efficiency, integration of neuroscience with psychology, and underlining challenges in terms of accessibility, standardization, and interdisciplinary collaboration. This visualization underlines the multidimensional impact of MBCT while highlighting areas for future research and intervention development.

### 4.7. Comparing Mindfulness-Based Cognitive Therapy (MBCT), Mindfulness-Based Stress Reduction (MBSR), and Congnitive Behavioral Therapy (CBT): Therapeutic Approaches and Mental Health Impact

MBSR and traditional CBT contribute to psychological well-being in various ways. While MBCT and MBSR train identical core mindfulness practices, their therapeutic focus and distinct outcomes differ, especially when compared to traditional CBT. First designed to prevent depressive relapses, MBCT integrates mindfulness techniques and cognitive behavioral strategies in a format that is particularly effective for participants suffering from recurrent depression. Based on their systematic review, researchers state that MBCT significantly reduces the risk of depressive relapses compared with usual care and is equally as effective as maintenance antidepressants. These findings underline one of the key strengths of MBCT in targeting the cognitive vulnerabilities linked with depression. At the same time, the more mechanistic but equally effective approach of CBT addresses negative thinking directly without the added component of mindfulness that acts to enhance emotional regulation.

CBT, or traditional CBT, is an older therapeutic intervention. It chiefly addresses maladaptive cognitions and behaviors through cognitive restructuring and behavioral activation. Researchers [99] performed a meta-analysis comparing mindfulness-based interventions against traditional CBT. Both practices reduce the symptoms of anxiety, depression, and stress; however, MBCT adds benefits by incorporating mindfulness, thus helping the individual be a non-judgmental observer of their thoughts. This is especially helpful for individuals high in cognitive reactivity, for whom confronting thoughts within CBT might be overwhelming.

In contrast, the development of MBSR was originally designed to reduce stress and increase general well-being, and thus, has been extensively adopted by many populations, either with or without major depression. Indeed, according to the review studies [135,139,144], MBSR remarkably reduces the level of stress and improves the quality of life for many conditions, such as chronic pain, cardiovascular disorders, and anxiety disorders. By nature, MBSR focuses less on mindfulness per se but rather on the cognitive restructuring elements of CBT or MBCT and is perhaps more effective simply in general stress management. However, in the study, researchers [35] noted that the integration of cognitive strategies in MBCT lends it to an advantage in specifically targeting anxiety symptoms linked to negative thought patterns, which is particularly beneficial for individuals with mood disorders.

Indeed, structural changes within the brain have been observed in MBCT and MBSR, particularly in the areas responsible for emotional control. However, this study [148] pointed out that, in addition to this, combining mindfulness with cognitive therapy components in MBCT resulted in extra benefits in cognitive functions such as attention and memory, which are also relevant for patients recovering from depression. Traditional CBT also influences cognitive functions but usually engages directly with cognitive distortions. By contrast, the mindfulness approach in MBCT encourages awareness of thoughts without needing immediate judgment and may, therefore, achieve longer-term cognitive gains. This study [101] underlined that the presence of a mindfulness element in MBCT not only develops cognitive efficiency but also helps build emotional resilience- an aspect less central in cognitive training within traditional CBT.

Specifically, the study found that ACT, another third-wave CBT incorporating mindfulness among its techniques, somewhat resembles MBCT, especially in its success in promoting subjective well-being. However, when dealing with recurrent depression, MBCT is outstanding because of its specific focus on preventing depressive relapses by combining mindfulness with cognitive restructuring.

Although MBSR is, because of its general focus on the reduction in stress and broad appeal, applied in many settings, MBCT applies a more specialized approach, which is typically clinical and aimed at the treatment of depression. As noted in the study [110], this structured approach of MBCT to address cognitive vulnerability for rumination and negative automatic thoughts brings considerable advantages over traditional CBT in those populations where such cognitive patterns are central to the disorder. The mindfulness component of MBCT adds a layer of emotional regulation that complements the cognitive restructuring central to CBT and may lead to deeper and more enduring therapeutic change.

A study [133] also showed that MBCT significantly enhanced subjective well-being in both clinical and non-clinical samples by emphasizing cognitive restructuring with an added emphasis on mindfulness. This contrasts with traditional CBT, which, although effective in improving well-being through altering negative thought patterns, perhaps does not offer the same emotional regulation and acceptance as mindfulness practices. On the other hand, whereas MBSR is effective in improving well-being, it is more oriented toward general stress management and mindfulness practices without the specific cognitive interventions provided in CBT and MBCT.

Researchers, in their study [71], reviewed the effectiveness of MBCT and MBSR in vascular disease-suffering individuals and found that though both interventions improve psychological and physical outcomes, the cognitive component of MBCT makes it particularly useful for managing depressive symptoms common in chronic illnesses. This, consequently, will suggest that integrating cognitive and mindfulness strategies in MBCT provides a holistic approach to dealing with mental and physical health challenges.

In a nutshell, MBCT and MBSR are similarly effective interventions, but given the different therapeutic emphases of traditional CBT, they suit different populations and psychological needs. This integration of cognitive therapy methods with mindfulness in MBCT imbues it with unique advantages in preventing depressive relapse and improving cognitive function in emotionally reactive people. Traditional CBT remains uniquely effective for those requiring structured cognitive restructuring, especially in cases of anxiety and depression. Being holistic, this all-encompassing approach to stress reduction in MBSR indeed faces more general applications across diverse populations, thus forming an excellent general enhancement of well-being. The systematic review has brought out the understanding of these differences, facilitating interventions custom-made to specific psychological conditions and therapeutic goals.

**Figure 8:**
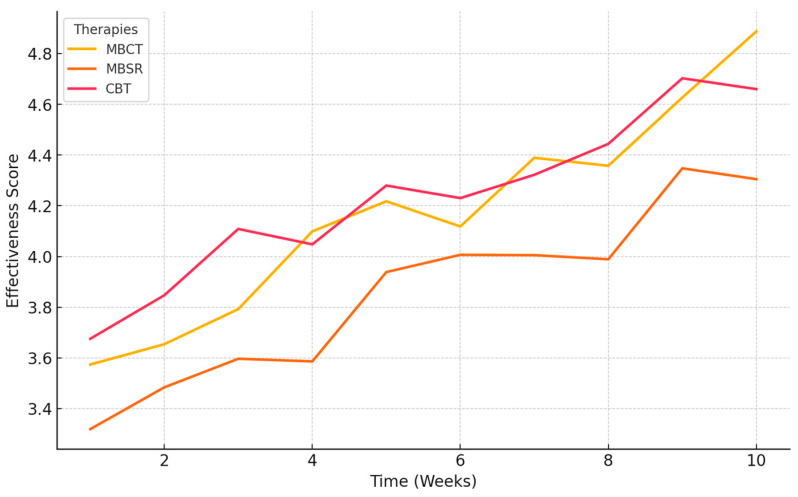
Trends in the effectiveness of mindfulness-based cognitive therapy (MBCT), mindfulness-based stress reduction (MBSR), and cognitive behavioral therapy (CBT) over time.

Finally, time trends in the effectiveness of MBCT, MBSR, and CBT point to unique strengths and clinical applications (Figure 8). MBCT reliably improves in treating depression and improving emotional regulation, probably because its integrated mindfulness and cognitive practices enhance neuroplasticity and resilience against depressive relapse. MBSR represents strong efficacy in stress management, reflecting its core design, though its impact on anxiety and depression is more gradual. The structured approach of CBT to cognitive restructuring and behavioral strategies promotes an overall robust, rapid practice in many domains, especially in the management of depression and anxiety. These findings underline the individual nature of interventions, but MBCT is especially good at emotional regulation, MBSR is particularly effective in stress reduction, and CBT is often quite versatile. Long-term neurobiological and psychological effects could be explored in future studies, incorporating elements from these therapies in different ways to see which way is most effective.

## 5. Discussion

### 5.1. Neuropsychological Insights into Mindfulness-Based Cognitive Therapy (MBCT): Impacts on Emotion, Cognition, and Well-Being

MBCT induced significant changes in the activation of the prefrontal cortex, amygdala, and anterior cingulate cortex, all brain regions that play a crucial role in emotional regulation. For example, the study [90] reported higher activity and connectivity in the prefrontal cortex after the MBCT treatment about enhanced executive function and decision-making. In the meantime, MBCT decreases activity within the amygdala, a brain region responsible for processing fear and emotional responses. Overall, such neuropsychological changes support the increased ability of MBCT patients to manage their emotions more successfully, which is mainly a great help for patients suffering from mood disorders. It also indicated that MBCT increases the activity connectivity with the anterior cingulate cortex and insula, enhancing the brain’s emotional balance.

Besides emotional regulation, MBCT also impacts cognitive functions, such as attention and memory, especially executive function. A study [148] highlighted that cognitive processes that are significantly enhanced include selective attention and working memory. These cognitive enhancement benefits are essential for the depressed individual to reduce cognitive distortions and improve overall cognitive performance. Furthermore, a study [112] reported that MBCT positively influences cognitive flexibility, which involves adapting to new information and dealing with complex tasks. In most individuals with depression, there is an impairment in this very aspect of cognitive flexibility; therefore, MBCT works effectively as an intervention in reinstating these critical cognitive functions.

The improvement in neuropsychological functions of the MBCT participants is highly associated with improvements in subjective well-being. Improvement in attention and memory, two critical neuropsychological factors in the study [91], strongly correlates with life satisfaction and overall happiness. This may indicate that due to MBCT, neuropsychological changes directly improve the individual’s subjective well-being and hence make MBCT an effective intervention for quality-of-life improvement. In this direction, the study [133] further extended the findings that MBCT significantly enhances subjective well-being, as expressed in higher scores on the Satisfaction with Life Scale (SWLS). This improvement in subjective well-being reflects the diminishment of negative symptoms and the enhancement of positive mental states, a core aim of MBCT.

Long-term studies provide further evidence for the durability of MBCT benefits. This evidence underlined that the cognitive benefits induced by MBCT, such as attention and memory, were preserved for an extended period after the intervention. This finding is particularly essential in chronic disorders like depression since sustained improvement is necessary to prevent relapses and maintain mental health. Besides, the study [35] also underlined that the MBCT effect on psychological health is long-lasting; the effects are maintained for extended periods. The fact that the impact of MBCT can be kept for a very long period underlines its potential as a durable intervention to sustain mental health and prevent the relapse of depression and anxiety.

As for the neuropsychological mechanisms of the effects of MBCT, it should be outlined that they are multi-factorial and complex and include both structural and functional changes in the brain. A study [94] investigated changes in attention regulation and emotional processing associated with MBCT in older adults, a population especially vulnerable to cognitive decline and emotional distress often accompanying aging. Their results indicate that MBCT significantly enhances these processes, promoting an improved capability to manage age-related cognitive decline and increase emotional well-being. Study [148] discussed how MBCT influences cognitive processes, including mindfulness, rumination, and meta-awareness, which are crucial for breaking the cycle of negative thinking characteristic of mood disorders. In this way, MBCT encourages greater mindfulness and reduces rumination, leading to more sustainable mental health improvements.

Further research by the study [113] suggests that MBCT is particularly effective in populations with bipolar disorder, where it has been shown to improve mood regulation and cognitive function without precipitating mania. This further demonstrates versatility in the application of MBCT against various mood disorders. Another review of studies [101] indicated mixed results on the effect of MBCT on cognitive outcomes for depression and suggested that, though generally effective, the degree of improvement in cognition may vary depending on the person and specific cognitive functions to be targeted. This is an encouragement toward more personalized approaches in MBCT interventions.

Such integration of neuropsychological findings into MBCT outcomes, evidenced in the systematic review studies, points out the profound impact of this therapy on the functioning and structure of the brain, touching emotional and cognitive functions. The latter neuropsychological changes are closely interlinked with improvements in subjective well-being, denoting MBCT as a functional therapeutic approach in both clinical and non-clinical settings. Such benefits being maintained in the long run further bolster the status of MBCT as a comprehensive mental health intervention. Further research in the future on these relationships, especially the neuropsychological ones in the long run, will go a long way towards maximizing the therapeutic utility of MBCT.

### 5.2. Challenges and Limitations

Notwithstanding the robust empirical support for the efficacy of MBCT in improving subjective well-being, some limitations must be pointed out. The most significant limitation is the uncertainty of the underlying neurobiological processes by which the improvement in mental health outcomes has been established. Although research suggests that MBCT causes neuroplasticity and improves cognitive and emotional regulation, the exact biological processes are more speculative. Future studies must utilize more sophisticated neuroimaging tools like fMRI and EEG to better understand the neural processes of MBCT. A further limitation relates to the assessment of personality traits and coping. As mentioned, these are highly vulnerable to demand characteristics and participant bias, as it is challenging to assume the “true” cognitive and affective change brought about by MBCT. Longitudinal designs involving more than one measure, where the change in such psychological variables is evaluated over long periods, are needed in future research to ascertain transient versus lasting effects.

Additionally, the efficacy of MBCT with varied populations is an issue that still needs to be resolved. While this review consolidates findings from several studies, cross-cultural investigations must be conducted to determine the generalizability of MBCT effects. Future studies need to determine whether the intervention works similarly well with culturally varied populations and whether modifications need to be made to maximize its effectiveness with non-Western populations. Moreover, the role of attention in mediating the effects of MBCT must be more extensively explored. While attentional control is considered a key component of mindfulness-based treatments, their definitive moderating impact on mental health improvement has not been officially investigated. Future studies must include multimodal assessment paradigms, such as behavioral tasks and neurophysiology, to outline how attentional mechanisms interact with MBCT training.

Lastly, combining MBCT with other therapies is a promising direction for its increased efficacy. Multimodal treatments combining MBCT and cognitive-behavioral therapy, pharmacotherapy, or biofeedback can yield synergistic effects. Additional research on sequential and concurrent application methods will offer valuable data on maximizing treatment effects for clinical and non-clinical groups. An ongoing, interdisciplinary research endeavor is needed to advance the theoretical underpinnings of MBCT, methodological rigor, and broaden its application to various populations. Correction of these deficiencies will allow for the creation of more targeted and effective mindfulness-based interventions, with the result of advancing a more nuanced understanding of the interface between mindfulness, cognition, and mental health.

### 5.3. Future Directions

Future research should focus on neuroimaging studies investigating causal relationships between MBCT and changes in brain function and structure. Longitudinal designs with multiple neuroimaging assessments before, during, and after MBCT interventions would clarify how neural connectivity and structural integrity evolve over time. Techniques such as functional magnetic resonance imaging (fMRI) and diffusion tensor imaging (DTI) can help assess the long-term impact of mindfulness training on the brain. Randomized controlled trials with neuroimaging features are needed to determine causality [156,157,158]. Subjects need to be randomized to MBCT, an active control treatment, or a waitlist control, with neuroimaging measures like fMRI, EEG, and MRS administered to compare neural activation and connectivity patterns between conditions. This would clarify the exact mechanisms through which MBCT affects emotional regulation and cognitive control [159,160,161,162].

Task-based fMRI can shed more light on the cognitive and emotional processing involvement of MBCT. Researchers can investigate changes in the prefrontal cortex, anterior cingulate cortex, amygdala, and insula activity by applying experimental paradigms such as emotion regulation tasks or attentional control tasks. Such results would help link neurobiological changes to self-reported mindfulness gains [163]. Resting-state functional connectivity MRI (rs-fMRI) must also be utilized to investigate the influence of MBCT on the large-scale brain networks, i.e., the default mode network (DMN), salience network, and executive control network. Dynamic causal modeling (DCM) or Granger causality analysis would reveal if MBCT directly influences the connectivity patterns related to cognitive and emotional control [164,165].

EEG and MEG would also investigate neural oscillations under mindfulness meditation, specifically in alpha, theta, and gamma frequency bands involved in attention and cognitive flexibility. A comparison of novice vs. experienced MBCT practitioners would ascertain whether specific oscillatory patterns predict long-term mindfulness benefits. Multimodal imaging techniques that combine fMRI, EEG, and MRS have the potential to give a broader picture of MBCT-related changes in the brain. The combination of structural MRI and fMRI would enable the evaluation of anatomical and functional changes. In contrast, MRI would quantify neurotransmitter changes, i.e., gamma-aminobutyric acid (GABA) and glutamate levels, involved in stress regulation and cognitive control. It is also necessary for future studies to address the role of MBCT in enhancing neuroplasticity [166,167]. Neuroplasticity in key regions such as the hippocampus, prefrontal cortex, and insula can be quantified by voxel-based morphometry (VBM) and cortical thickness measurements. It would determine whether MBCT exerts neuroprotective effects that underline resilience against stress and affective disorders [168,169,170].

Using such neuroimaging techniques, the research may extend beyond correlation and to causation, with greater insight into the impact of MBCT on brain structure and activity in mental health promotion [171]. The use of multimodal imaging and causal modeling techniques will determine the specific neural mechanisms through which MBCT has its therapeutic influences, enabling mindfulness-based interventions to be refined for both clinical and non-clinical samples [172,173,174].

## 6. Conclusions

To sum up, the systematic review comprehensively demonstrates the robust efficacy of MBCT in enhancing subjective well-being through improved neuropsychological outcomes. MBCT constantly decreases symptoms related to depression, anxiety, and stress while appreciably enhancing cognitive functions and emotional regulation in several populations. These findings support the potential of MBCT in fostering neuroplastic changes and hence, providing long-term intervention for the management of mental health. Further high-quality research is needed to better understand the long-term benefits and underlying neurobiological mechanisms of MBCT. This review on the critical integration of psychology and neuroscience in furthering the understanding and application of mindfulness-based interventions in mental health care will lay the ground for optimizing therapeutic outcomes and developing a trail of lasting improvements in mental health across populations.

## Abbreviations

The following abbreviations are used in this manuscript:

ACT	Acceptance and Commitment Therapy
APA	American Psychological Association
BPS	Biopsychosocial
BPSD	Behavioral and Psychological Symptoms of Dementia
CBT	Cognitive Behavioral Therapy
CRF	Cancer-Related Fatigue
CRP	C-reactive protein
DMN	Default Mode Network
DSM	Diagnostic and Statistical Manual of Mental Disorders
EEG	Electroencephalography
fMRI	Functional Magnetic Resonance Imaging
HPA	Hypothalamic-Pituitary-Adrenal (axis)
HRV	Heart Rate Variability
ICD	International Classification of Diseases
IL-6	Interleukin 6
ITT	Intent-to-Treat
MBT	Mindfulness-Based Therapy
MBCT	Mindfulness-Based Cognitive Therapy
MBI	Mindfulness-Based Interventions
MBSR	Mindfulness-Based Stress Reduction
MCI	Mild Cognitive Impairment
MRI	Magnetic Resonance Imaging
MS	Multiple Sclerosis
OFC	Orbitofrontal Cortex
PFC	Prefrontal Cortex
PRISMA	Preferred Reporting Items for Systematic Reviews and Meta-Analyses
PTSD	Post-Traumatic Stress Disorder
PwD	People with Dementia
QoL	Quality of Life
RCT	Randomized Controlled Trial
SRMA	Systematic Review and Meta-Analysis
UTs	Uncontrolled Trials
SR	Systematic Review
PilS	Pilot Studies
NRCTs	Non-Randomized Controlled Trials
QED	Quasi-Experimental Design
RPGST	Randomized Parallel-Group Superiority Trial
PPIDs	Pre-Post Intervention Designs
ES	Empirical Studies
QS	Qualitative Studies
CMA	Controlled Mediation Analyses
SC	Self-Compassion
SoC	State of Comparison
SMD	Standardized Mean Difference
SWB	Subjective Well-Being
